# Pancreatectomy with En Bloc Superior Mesenteric Vein and All Its Tributaries Resection without PV/SMV Reconstruction for “Low” Locally Advanced Pancreatic Head Cancer

**DOI:** 10.3390/cancers16122234

**Published:** 2024-06-15

**Authors:** Viacheslav Egorov, Pavel Kim, Soslan Dzigasov, Eugeny Kondratiev, Alexander Sorokin, Alexey Kolygin, Mikhail Vyborniy, Grigoriy Bolshakov, Pavel Popov, Anna Demchenkova, Tatiana Dakhtler

**Affiliations:** 1 Ilyinskaya Hospital, 143421 Moscow, Russia; drpoul@yandex.ru (P.K.); s.dzigasov@ihospital.ru (S.D.); evgenykondratiev@gmail.com (E.K.); koligin@gmail.com (A.K.); m.vyborniy@ihospital.ru (M.V.); gregbol19912020@gmail.com (G.B.); popovpavel@mail.ru (P.P.); demchenkovaanna89@gmail.com (A.D.); tdakhtler@mail.ru (T.D.); 2 Burnasyan State Research Center of the Federal Medical Biological Agency, 119435 Moscow, Russia; 3 Radiology Department, Vishnevsky National Medical Research Center of Surgery, 117997 Moscow, Russia; 4 Department of Mathematical Methods in Economics, Plekhanov Russian University of Economics, 117997 Moscow, Russia; alsorokin@statmethods.ru

**Keywords:** locally advanced PDAC, vein resection without PV/SMV reconstruction, pancreatic cancer, portal venous collaterals, low pancreatic cancer, superior mesenteric vein occlusion, CT-based venous reconstructions

## Abstract

**Simple Summary:**

Radical pancreatectomies with superior mesenteric vein (SMV) resection without portal-to-superior mesenteric vein (PV/SMV) reconstruction are scarcely discussed in the literature. The existing data contain around 15 cases published with unclear oncological results and algorithms of patients’ selection. In the present report, we analyzed the short- and long-term results of 19 consecutive pancreatectomies with SMV resection without PV/SMV reconstruction for locally advanced pancreatic ductal adenocarcinoma, and discussed the role of CT-based preoperative reconstructions and selection criteria for radical and safe surgery in this highly specific group of patients.

**Abstract:**

The “vein definition” for locally advanced pancreatic ductal adenocarcinoma (LA PDAC) assumes portal-to-superior mesenteric vein (PV/SMV) unreconstructability due to tumor involvement or occlusion. Radical pancreatectomies with SMV resection without PV/SMV reconstruction are scarcely discussed in the literature. Retrospective analysis of 19 radical pancreatectomies for “low” LA PDAC with SMV and all its tributaries resection without PV/SMV reconstruction has shown zero mortality; overall morbidity—56%; Dindo–Clavien—3–10.5%; R0—rate—82%; mean operative procedure time—355 ± 154 min; mean blood loss—330 ± 170 mL; delayed gastric emptying—25%; and clinically relevant postoperative pancreatic fistula—8%. In three cases, surgery was associated with superior mesenteric (n2) and common hepatic artery (n1) resection. Surgery was completed without vein reconstruction (n13) and with inferior mesenteric-to-splenic anastomosis (n6). There were no cases of liver, gastric, or intestinal ischemia. A specific complication of the SMV resection without reconstruction was 2–3 days-long intestinal edema (48%). Median overall survival was 25 months, and median progression-free survival was 18 months. All the relapses, except two, were distant. The possibility of successful SMV resection without PV/SMV reconstruction can be predicted before surgery by CT-based reconstructions. The mandatory anatomical conditions for the procedure were as follows: (1) preserved SMV-SV confluence; (2) occluded SMV for any reason (tumor or thrombus); (3) well-developed inferior mesenteric vein collaterals with dilated intestinal veins; (4) no right-sided vein collaterals; and (5) no varices in the upper abdomen. Conclusion: “Low” LA PDACs involving SMV with all its tributaries can be radically and safely resected in highly and specifically selected cases without PV/SMV reconstruction with an acceptable survival rate.

## 1. Introduction

Pancreatic ductal adenocarcinoma (PDAC) is one of the leading causes of cancer deaths worldwide, with substantially increasing prevalence during the past twenty years [1,2]. The relative 5-year survival rate for all stages of PDAC is around 12%, being better for non-metastatic forms (40–45% of patients): 38.8% for localized and 14.9% for locally advanced (LA) PDAC [3]. Better survival achieved during the last two decades is based on advances in perioperative cytotoxic chemotherapy and more aggressive surgical approaches. Due to studies showing equal survival after pancreatectomy (PE) with venous resection compared to patients after standard PE, and studies demonstrating better survival after PE with vein resection compared to conservative treatment [4,5,6,7,8], resections of the superior mesenteric (SMV) and portal veins (PV) with reconstruction have become an accepted practice for borderline resectable PDAC since the beginning of the century.

Currently, PDAC with SMV and/or PV involvement of more than 180 degrees is considered borderline resectable if the SMV-PV flow can be restored. If the PV-SMV route is not reconstructable after PE, this cancer is considered locally advanced [9]. LA PDAC comprises approximately 30% of PDACs with a 5-year survival rate of 12–40% in surgical series [10,11,12,13], although that series presented patients with cancers united by the “arterial” and not the “venous” definition of the LA PDAC.

Only a few case reports on PE with resection of SMV with all the tributaries are presented in the literature [14,15,16,17,18], with 11 PDAC patients in total. We presented our experience of 19 cases of resection of SMV with all its tributaries without SMV-PV reconstruction. The feasibility and safety of such procedures are strictly dependent on the preoperative assessment of the venous anatomy and intraoperative estimation of the venous collaterals function.

## 2. Patients and Methods

Current NCCN guidelines definitions of borderline resectable and LA PDAC are based on the tumor’s relationship with surrounding vessels [9]. The crucial surrounding vessels are the celiac trunk (CT), superior mesenteric artery (SMA), common hepatic artery (CHA), and the SMV-PV route. Concerning the SMV/PV involvement or thrombosis, guidelines define borderline resectable cancer with an option of vascular reconstruction after venous resection (“venous definition for borderline resectable PDAC”) and locally advanced disease with “unreconstructable SMV/PV due to tumor involvement or occlusion” (“venous definition for LA PDAC”). 

Nineteen consecutive patients with pancreatic head ductal adenocarcinoma underwent SMV resection with all its tributaries without PV-SMV reconstruction in addition to partial or total pancreatectomy from November 2006 to December 2023. Demographic and perioperative data of all the patients were retrospectively explored from medical records, follow-up charts, and radiological reports. All the patients with thrombosis/involvement of the SMV and all its tributaries were discussed at multidisciplinary meetings, and all the procedures were undertaken to perform R0-resection. The resectability assessment was based on NCCN definitions, imaging data, and the surgeon’s experience. From 2006 to 2018, PE with excision of the SMV and all the tributaries due to the well-developed left-sided collaterals was considered a borderline rather than unresectable situation in our department. Because of this, before 2019, patients received 6 cycles of neoadjuvant treatment, and since 2020, all the patients were operated on after 12 cycles of chemotherapy. Chemoradiation was not used.

PDAC was confirmed by biopsy before the beginning of treatment in all cases. The following guidelines were used for the description of peri- and postoperative data: Clavien–Dindo classification—for the postoperative morbidity with Grade ≤ 2 for minor, and Grade ≥ 3A for major complications [19]; International Study Group on Pancreatic Fistula classification—for postoperative pancreatic fistula (POPF) [20]; International Study Group of Pancreatic Surgery (ISGPS) classification—for post-pancreatectomy hemorrhage (PPH) [21]; the Royal College of Pathologists classification—for R0–R1 definitions [22]; the College of American Pathologists classification—for the assessment of the grade of posttreatment tumor regression [23]. Ischemic morbidity was defined as an abdominal organ complication(s) caused by surgery-related ischemia. Tumor size was measured in mm using computer tomography before surgery and at pathohistological examination after surgery. 

We considered pancreatic head cancer as “low” when the tumor was located in the uncinate process or lower part of the pancreatic head. Complications, readmissions, and mortality were registered up to the 90th postoperative day. Overall (OS) and progression-free (PFS) survival were measured from the date of tissue diagnosis until death [9,24]. Survival rates were determined based on the last CT or MRI results (PFS), last visit to the hospital, or follow-up phone calls. The last data collection was performed in March 2024.

### 2.1. Radiology 

**Figure 1:**
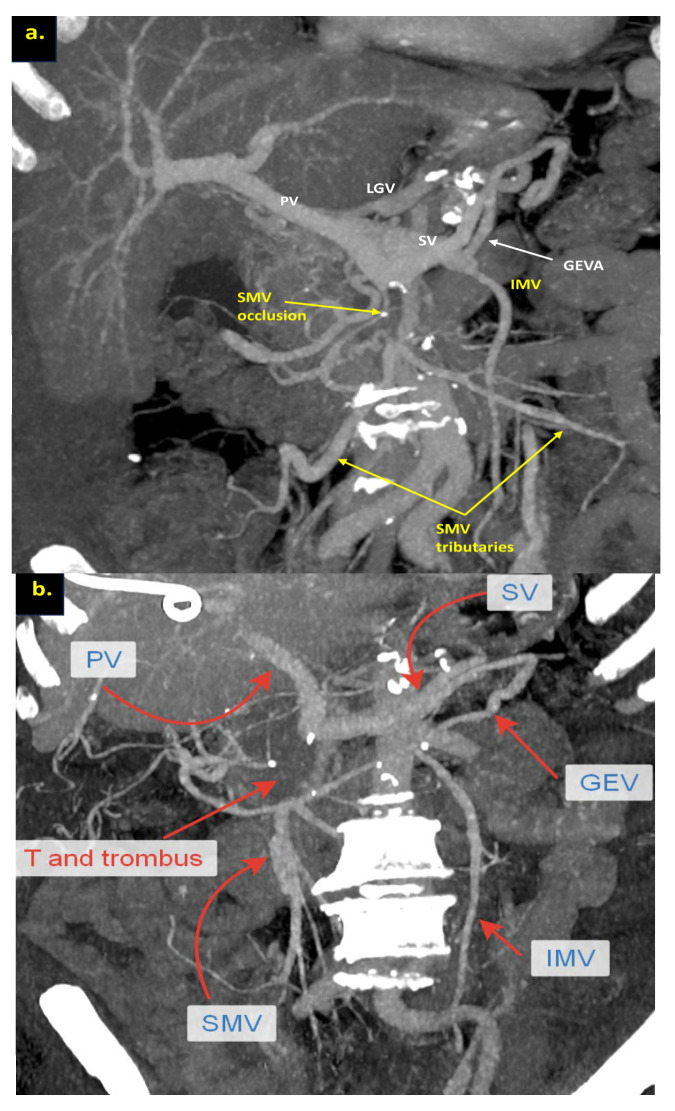

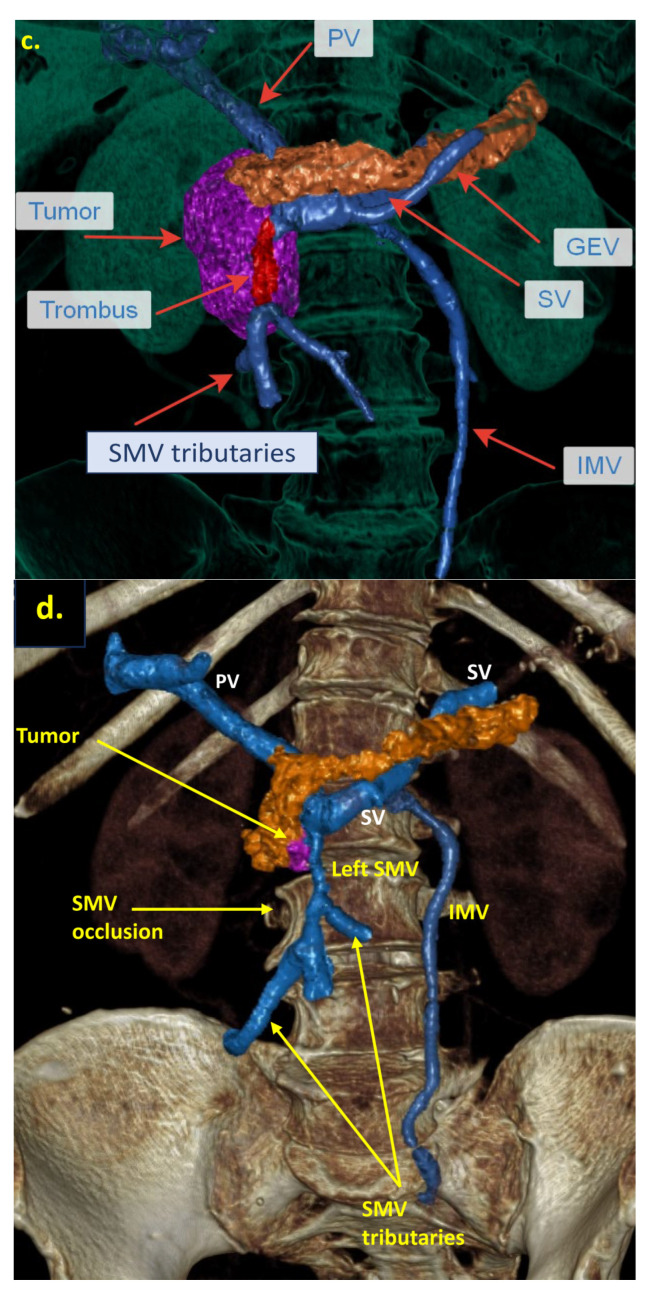
Post-processing reconstructions of MDCT portal phase images for adenocarcinomas of the pancreatic uncinate process, associated with SMV occlusion, caused by its thrombosis and/or involvement. Three-dimensional maximum intensity projections (MIP) and volume-rendered (VR) imaging show preserved SMV-SV confluence, occluded SMV, well-developed IMV collaterals with dilated intestinal veins, and the absence of right-sided vein collaterals and varices. (**a**) Three-dimensional MIP reconstruction shows the dilated SMV tributaries, gastro-epiploic venous arcade (GEVA), left gastric (LGV), and inferior mesenteric veins (IMV) flowing into the splenic vein (SV); (**b**) three-dimensional MIP reconstruction. Dilated gastro-epiploic vein (GEV), and IMV anastomosing with the SV; (**c**) three-dimensional VR reconstruction. SMV is thrombosed and surrounded by the tumor; (**d**) three-dimensional VR reconstruction shows the dilated SMV tributaries and IMV flowing into the splenic vein (SV). The tumor shrank after chemotherapy, the SMV is occluded and the narrow left SMV is going along it. PV-portal vein. Abbreviations correspond to all the figures.

The combination of CT, MRI, PET/CT, and blood CA 19-9 was used as the best modality for the selection of candidates for radical surgery in patients with LA PDAC [11] by the evaluation of vascular involvement and distant metastases. Interpretation of the standardized digital images from the preoperative multiphase pancreatic MDCTs, MRIs, and PET/CTs (when available) of all 19 patients was independently performed by three abdominal image readers (PP, AD, EK). High-quality CT-based pancreatic protocol was the standard method of the anatomical resectability assessment with the analysis of four phases: native, arterial (pancreatic), portal venous, and delayed phases following high concentrated (350–400 mg/mL) non-ionic iodinated contrast media injection at the rate of 2.5–5.0 mL/s. CT examinations were performed between 2006 and 2024, using 32- to 256-detector scanners Philips Ingenuity, Philips iCT, GE optima CT 540, Philips Brilliance CT, Philips iCT, Toshiba Aqullion, and GE Revolution, with machine-specific image acquisition ranging from 0.625 to 3 mm axial slice thickness. Since 2008, 3D abdominal vein reconstructions have become useful and necessary components for decision-making on possible radical surgery for “low” pancreatic cancers with SMV thrombosis/involvement (Figure 1a–d). After ruling out the distant metastases, only patients with specific vein anatomy can be candidates for radical surgery. The mandatory anatomical conditions for the procedure were the following: (1) preserved SMV-SV confluence; (2) occluded SMV for any reason (tumor or thrombus); (3) well-developed inferior mesenteric vein (IMV) collaterals with dilated intestinal veins; (4) no right-sided vein collaterals (which will be sacrificed); and (5) no varices in the upper abdomen.

### 2.2. Surgery

Significant decreasing or normalization of the serum CA 19-9 (Table S1) were the important criteria for patients’ selection for surgery. One patient was a non-secretor. After ruling out the metastases by laparoscopy, the procedures were followed by laparotomy. Intraoperative ultrasound (IOUS) and frozen-section biopsy were widely used in any suspicious circumstances found at surgery. 

All the tributaries of the SMV, which were planned to be resected, were visualized and clamped for 30 min using vascular instruments. The adequacy of the collateral flow to the IMV was assessed at a 30-min occlusion test: the test was considered “positive” and resection without PV-SMV reconstruction—safe in patients with no or mild edema of the small intestine without or with a short-term (during the test time) color change. If only the short (up to 30 cm) proximal jejunum segment was congestive, it could be safely resected. If the intestine looked slightly edematous after 30 min of clamping and its color was normal, we considered it safe to proceed with radical surgery. In cases with longer venous congestion, especially with a changing of the color of the intestine to cyanotic, it was reasonable to stop with radical surgery. During the occlusion test, the adequacy of the collateral flow to the liver was assessed using Doppler IOUS measurements from the liver parenchyma. Detection of the adequate portal intraparenchymal flow velocity ≥ 12 cm/s after clamping of the SMV tributaries and a “positive” intestinal occlusion test were considered sufficient conditions for resection without PV-SMV reconstruction.

**Figure 2:**
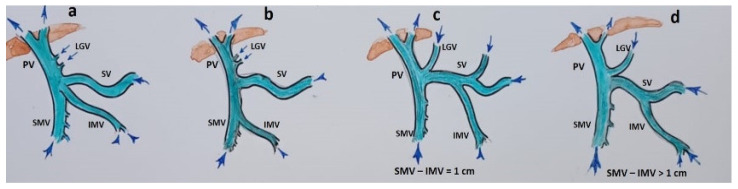
Anatomical variants of the inferior mesenteric vein (IMV) junctions with the spleno-mesenteric system. (**a**) IMV flows into the SMV-SV angle; (**b**) IMV flows into the SMV; (**c**,**d**) IMV flows into the SV.

**Figure 3:**
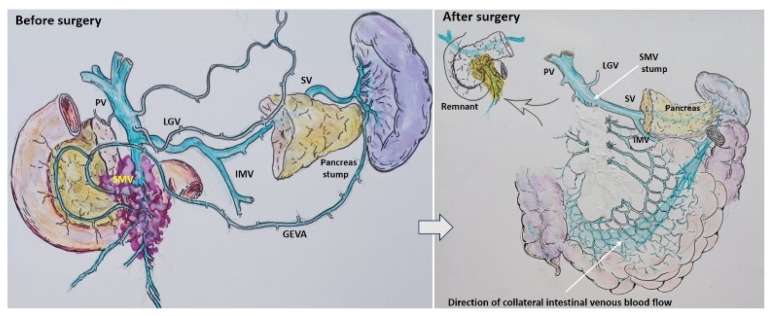
Scheme. Scenario 1: Pancreatoduodenectomy or total pancreatectomy with the SMV and all its tributaries resection without reconstruction.

After a favorable decision on resectability, two scenarios were possible, depending on the patient’s anatomy (Figure 2) and tumor spread on IMV. Scenario 1: if the IMV was not involved in the tumor, the SMV with all its tributaries was resected without reconstruction (Figure 3). 

**Figure 4:**
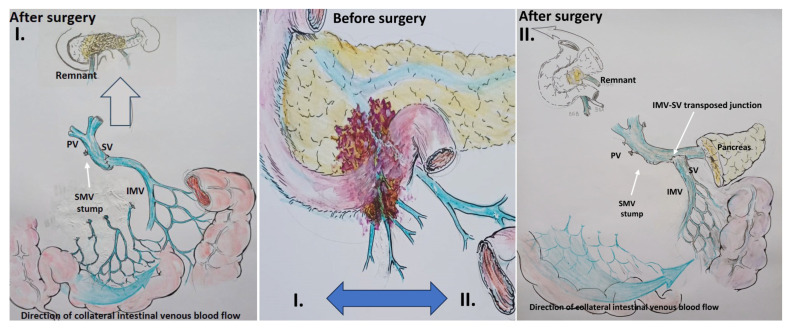
Scheme. Scenario 2: Total pancreatectomy (**right**) and pancreatoduodenectomy (**left**) with the SMV and all its tributaries resection with IMV-SV anastomosis.

Scenario 2: If the IMV was involved, it was resected and replanted in the splenic vein end-to-end in the cases of total pancreatectomy, or end-to-side in the case of pancreatoduodenectomy (Figure 4). 

The superior mesenteric artery in two cases and the common hepatic artery (Michels IX anatomy) in one case were also resected and reconstructed for the achievement of the R0 procedure. The Cattell–Braasch maneuver was used when combined vein and arterial resections were expected.

**Figure 5:**
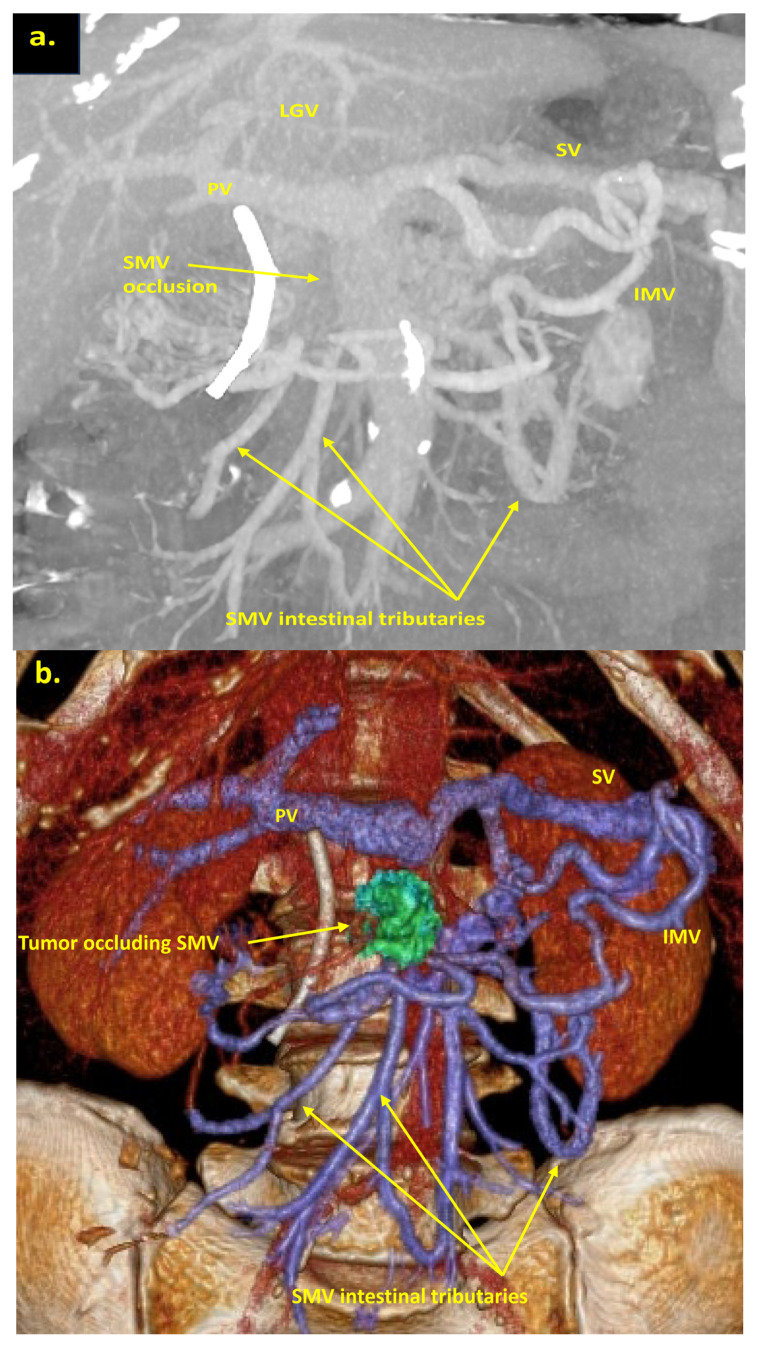

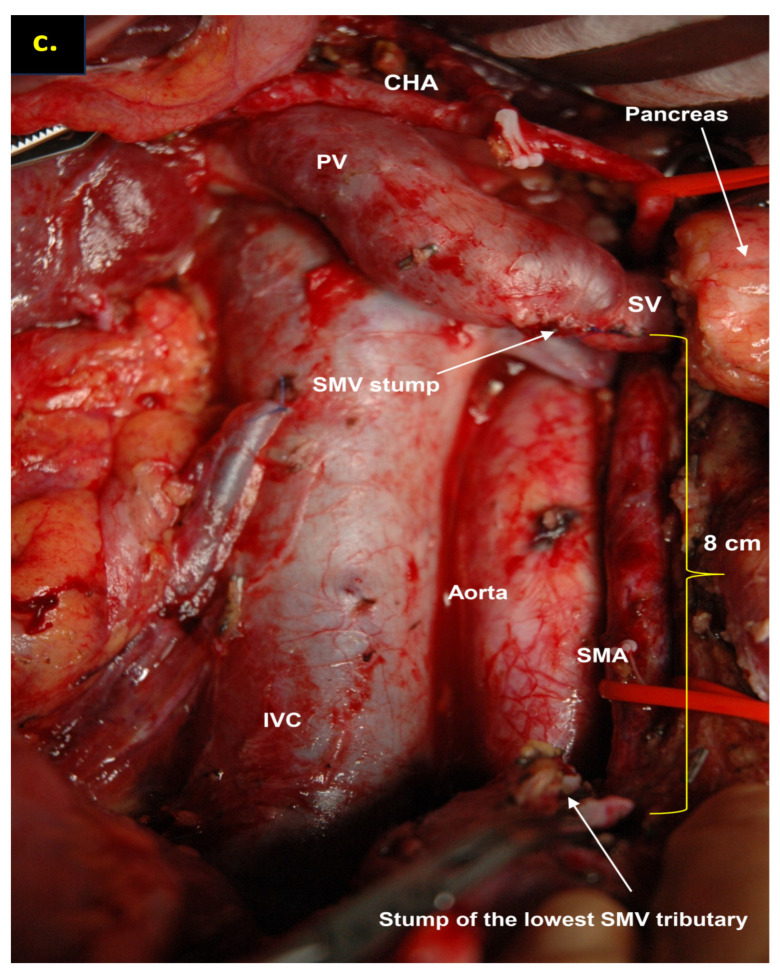
Pancreatoduodenectomy with the SMV and all its tributaries resection without reconstruction for the uncinate PDAC in 64-year-old male. On CT the tumor invades the SMV, which is occluded, the SMV-SV confluence is preserved, and the IMV collaterals are well-developed with dilated intestinal veins, without right-sided vein collaterals and varices. (**a**) Three-dimensional MIP and (**b**) three-dimensional VR reconstructions show the dilated SMV tributaries, gastro-epiploic venous arcade, and IMV flowing into the SV; (**c**) the picture of the operating field after the extended Whipple procedure with the SMV and all its tributaries resection without reconstruction. CHA—common hepatic artery, IVC—inferior vena cava.

**Figure 6:**
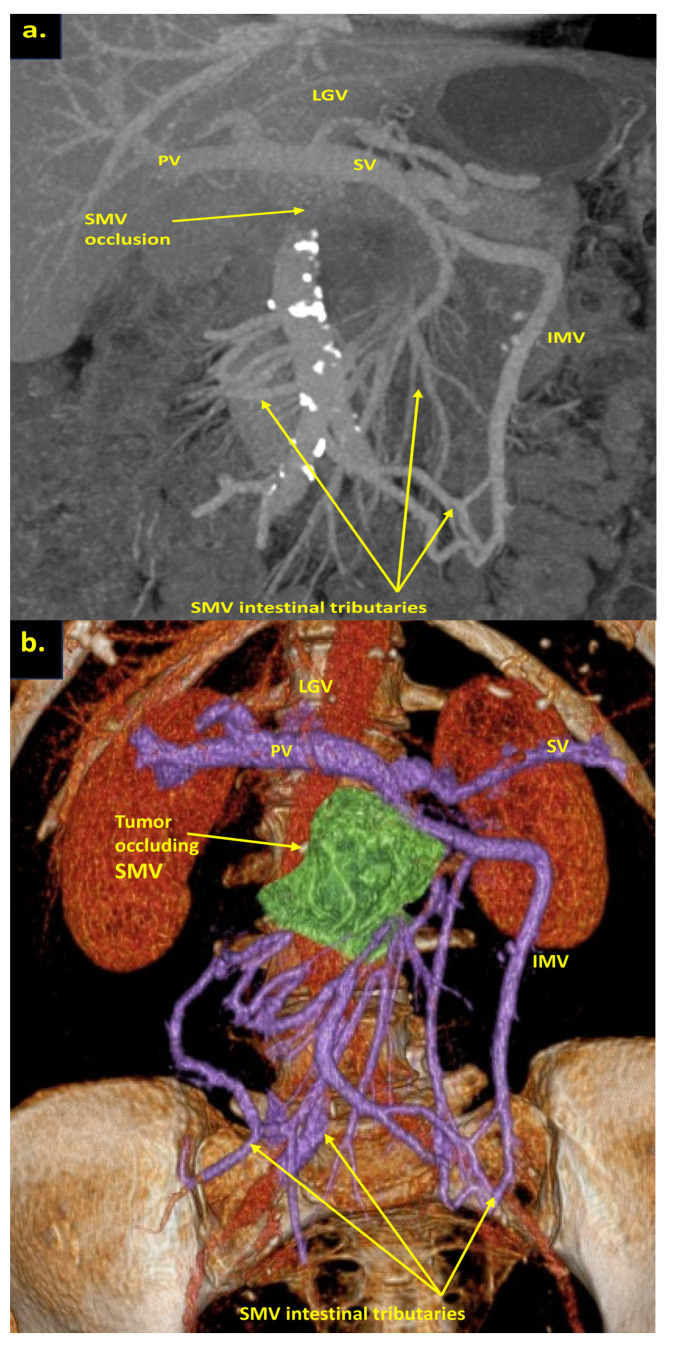

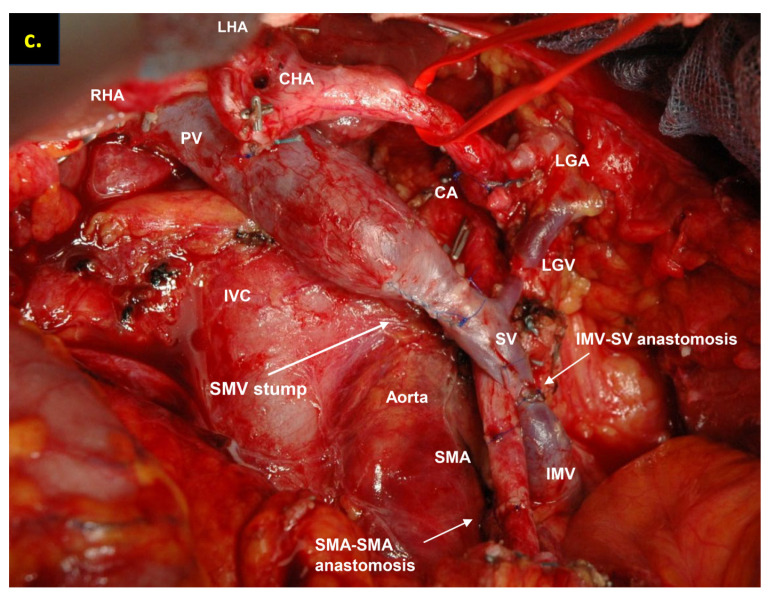
Total pancreatectomy with the SMV and all its tributaries resection with resection of IMV and its transposition into SV stump, combined with SMA resection and reconstruction for the uncinate PDAC in 71-year-old female. On CT the tumor invades the IMV and SMV (the last one is occluded), the SMV-SV confluence is preserved, and the IMV collaterals are well-developed with dilated intestinal veins, without right-sided vein collaterals and varices. (**a**) Three-dimensional MIP and (**b**) three-dimensional VR reconstructions show the dilated intestinal veins flowing into the IMV, which connects to the SV; (**c**) the picture of the operating field after the extended Whipple procedure with the SMV and all its tributaries resection without reconstruction. CHA—common hepatic, LGA—left gastric, SA—splenic, RHA—right hepatic, LHA—left hepatic, SMA—superior mesenteric arteries, CA—celiac artery, IVC—inferior vena cava.

**Figure 7:**
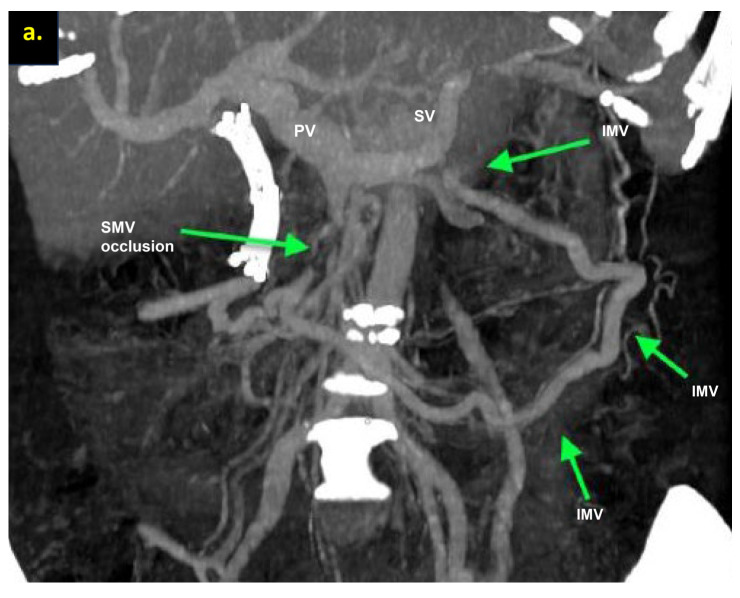

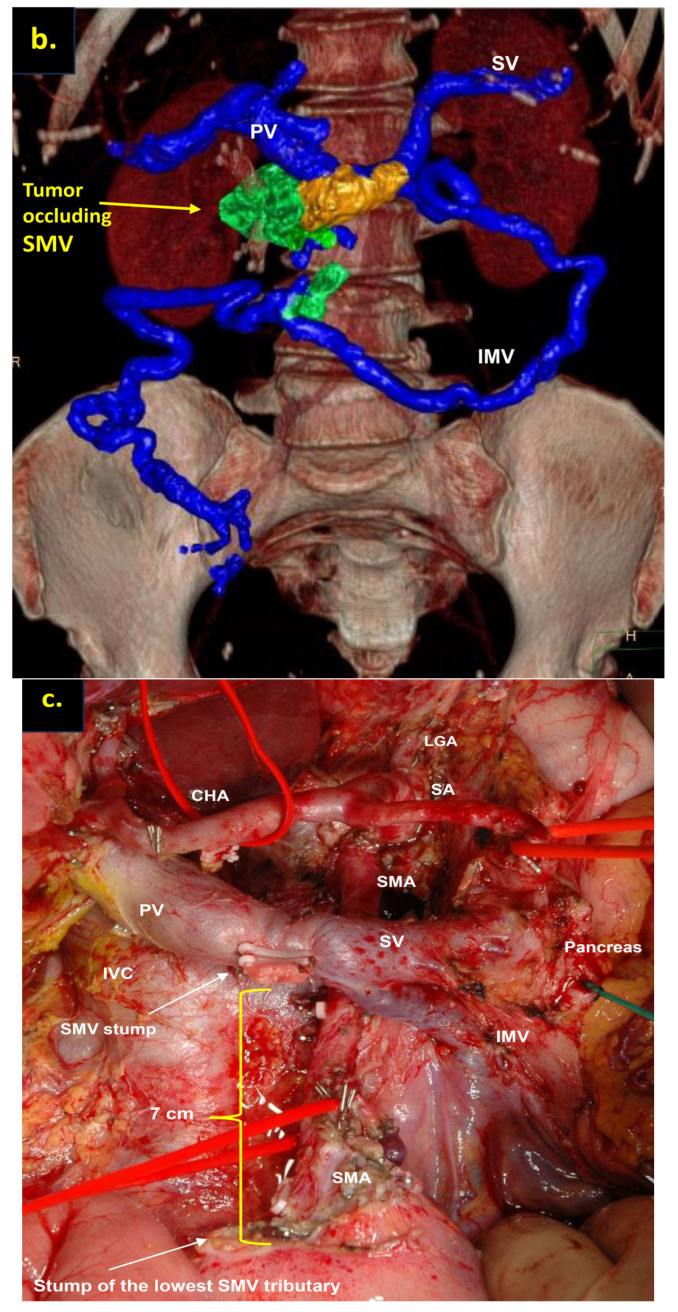

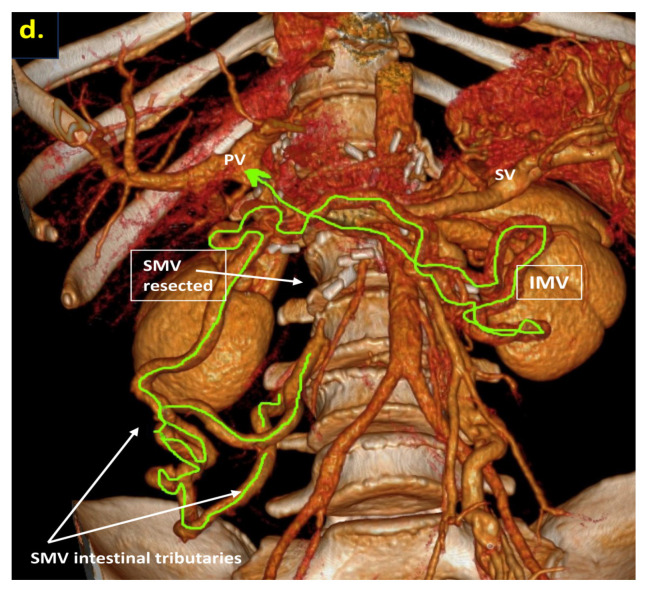
Pancreatoduodenectomy with the SMV and all its tributaries resection without reconstruction for the uncinate PDAC in 57-year-old female. On CT the tumor invades the SMV, which is occluded, the SMV-SV confluence is preserved, and the IMV collaterals are well-developed with dilated large collecting intestinal vein, without right-sided vein collaterals and varices. (**a**) Three-dimensional MIP and (**b**) three-dimensional VR reconstructions show the dilated large collecting intestinal vein flowing into the IMV, which connects to the SV; (**c**) the picture of the operating field after the extended Whipple procedure with the SMV and all its tributaries resection without reconstruction. CHA—common hepatic, LGA—left gastric, SA—splenic, SMA—superior mesenteric arteries, IVC—inferior vena cava; (**d**) the way of the intestinal blood flow after surgery.

Partial or total pancreatectomy was performed with extended lymph node dissection (extended pancreatectomy), which included the removal of lymph nodes of group 16 a,b (Figure 5) and/or extended retroperitoneal periaortal dissection during vascular resections (Figure 6) or with standard lymph node dissection (Figure 7), if there were no suspicious lymph nodes detected on imaging before surgery. Extension of the resection of the proper pancreas (body or partly tail as an addition to the head removal) did not mean extended pancreatectomy. 

In this study, only cases with full excision of SMV trunk and resection of all its tributaries, namely middle and right colic veins, ileocolic trunk, and all the first-order SMV branches (jejunal and ileal), without the reconstruction of above-mentioned vessels, were included. The usual length of the resected SMV with the branches in these cases was 7–8 cm (Figure 5 and Figure 7).

Ultrasonography was performed for the first three postoperative days. Liver function tests (ALT, AST, bilirubin, albumin, GGTP) were taken on postoperative days 1, 2, 7, and 14. Patients with soft pancreas received somatostatin inhibitors percutaneously 100–200 mkg three times daily for seven days postoperatively, and all patients received proton pump inhibitors for three months. The drains were removed between postoperative days 12 and 31. Patients were discharged with a drain production of around 500 mL of serous fluid. Follow-up consisted of physical examination, blood tests, including CA-19-9, and CT imaging at 3-month intervals for the first two years, and at 6-month intervals after the second postsurgical year. By the end of March 2024, no patients were lost to follow-up.

### 2.3. Statistics 

The study results were analyzed with the use of IBM SPSS Statistics 27 Software (IBM, Armonk, NY, USA). For quantitative indicators, parametric statistics were used as measures of descriptive statistics: arithmetic average, standard deviation, minimum and maximum value, and distribution quartile for non-parametric statistics. A comparison of quantitative indicators between groups was performed using a parametric Student’s t-test. Data distribution normality was assessed using Shapiro–Wilk’s test. The results of the analysis are presented using parametric statistics in the form of M ± SD and for non-parametric statistics in the form of Me [Q1; Q3]. Quantitative and qualitative paired data were compared using the Wilcoxon test, whereas unpaired quantitative and qualitative data were compared using the Mann–Whitney U test and Fisher’s exact test, respectively. The relationship between quantitative indicators was analyzed by the scattering diagrams and paired correlation coefficients (parametric Pearson and non-parametric Spearman coefficients). Survival analysis for overall (OS) and progression-free survival (PFS) was carried out by the Kaplan–Meier method. The median survival time is presented as median and 95% confidence interval in months: Me [95% CI: Me1; Me2]. Survival in groups was compared using a long-rank criterion. The critical level of significance was set at *p* < 0.05.

## 3. Results 

### 3.1. Patient Characteristics and Outcomes 

Among 903 pancreatic resections performed from January 2006 to December 2023, 299 (33%) were accompanied by vein resections, and 19 of them by SMV resection with all its tributaries without PV-SMV reconstruction (2%). All the procedures were performed after Gemcytabine- or FOLFIRINOX-based neoadjuvant chemotherapy for PDAC in 16 patients with Stage cT3NxM0 and in 3 patients with Stage cT4NxMo (UICC, 8th edition) [25]. 

Results for the patients with T3 (only veins involvement) and T4 (associated arterial involvement) were analyzed and presented separately. In two cases, narrow additional SMV was found on the preoperative CT and at surgery. 

### 3.2. T3 Group Results 

Demographic, clinical, and perioperative data of patients with T3 are summarized in Table 1, Table 2, and Table S1. The mean age of patients was 62 ± 8 years (range 39–74), surgery time was 401 ± 75 min (range 195–475), and blood loss was 346 ± 168 mL (range 100–550 mL). Length of hospital stay was 13.6 ± 3.9 days. There were no R2-resections with an 81% R0-resection rate. In this group, we performed pancreatoduodenectomy more than twice as often compared to the total PE. Surgery without vein reconstruction was performed more than twice as often as with IMV-SV reconstructions. Four IMV-SV reconstructions were performed during the total PE and only once during the Whipple procedure. In 56% of cases, there were no metastases in removed lymph nodes.

There were no deaths, postoperative bleeding, blood transfusions, or gastric, bowel, or liver ischemia. Postoperative serum ALT/AST > 250 U/l was observed in 11 cases (max 2140 U/l), and all the patients with IMV-SV reconstructions had aminotransferase elevation. ALT/AST levels significantly decreased or normalized after 3–5 postoperative days in all cases. In all patients except two, the pancreatic remnant was fibrotic and hard with a dilated duct, and Grade B POPF developed in only one patient who was discharged with the drain. One patient developed a severe complication—bile leakage on the fifth postoperative day, which was successfully treated by relaparotomy and additional draining (#12, Table S1). The most frequent complication was lymphorrhea (44%) and 2–3 days-long small bowel edema (31%), but neither event influenced the patients’ condition or length of stay.

### 3.3. Long-Term Outcomes after Surgery for Patients with Stage cT3NxM0

**Figure 8:**
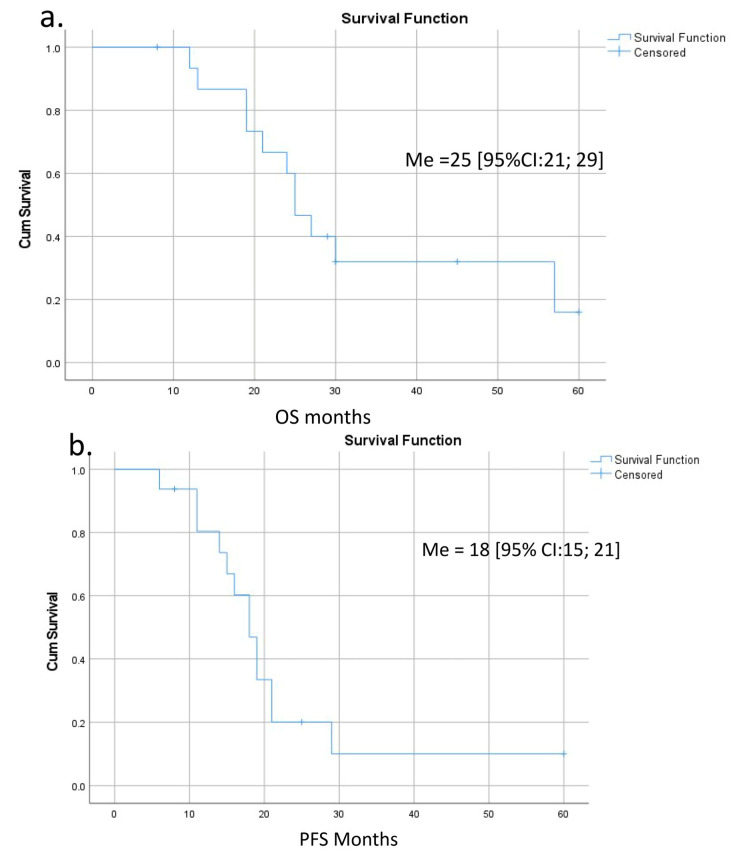

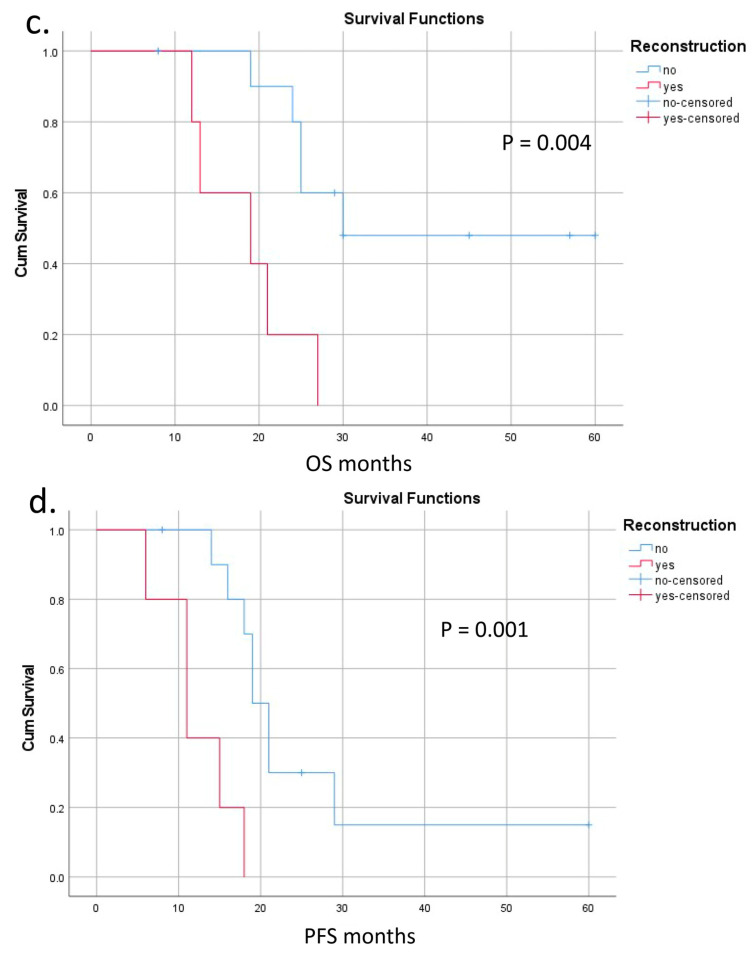

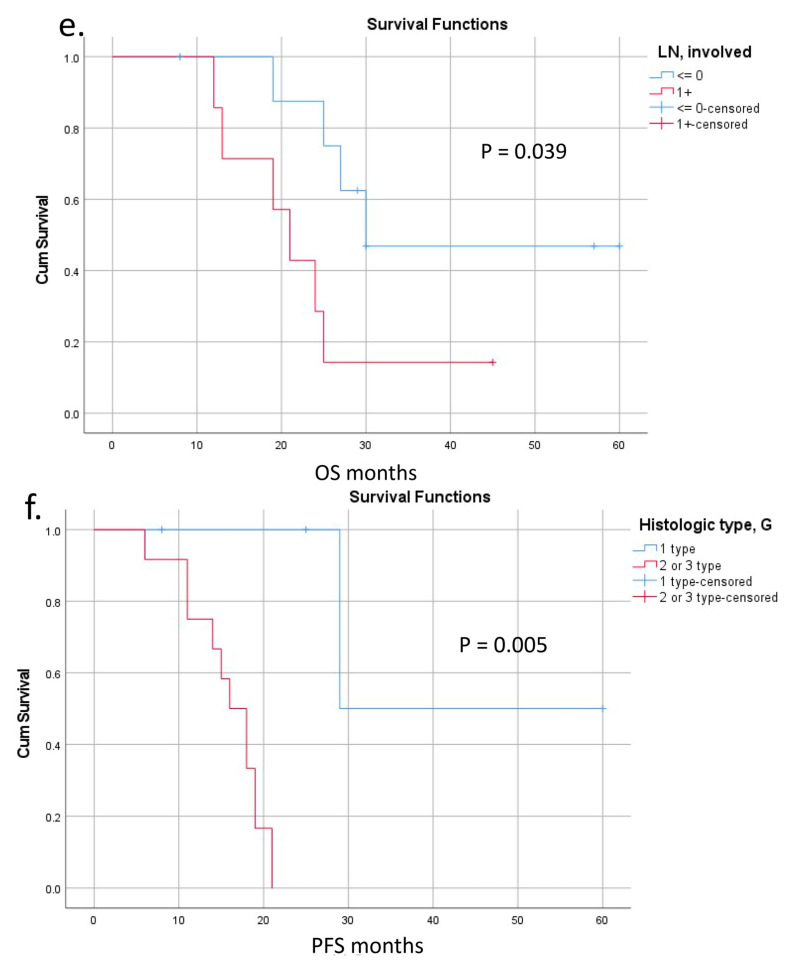
Overall (OS) and progression-free (PFS) survival after pancreatectomies with the SMV and all its tributaries resections without PV-SMV reconstructions. (**a**) The median overall survival; (**b**) the progression-free survival; (**c**,**d**) OS and PFS were significantly dependent on the presence (red line) or absence (blue line) of the IMV-SV reconstructions during pancreatectomy. IMV-SV reconstructions were associated with worse survival; (**e**) overall survival inversely depended on lymph node involvement; it was significantly better if cases with N-(blue line) compared to N+ (red line); (**f**) tumor grade was inversely related to the PFS, and survival was significantly better in patients with well-differentiated tumors (blue line).

Survival was calculated for 15 patients with a follow-up period of 32 [95% CI: 21; 37] months (Figure 8 and Table S2). The last case was excluded because of the short time of follow-up. OS and PFS were 25 [95% CI: 21; 29] and 18 [95% CI: 15; 21] months, respectively (Figure 8a,b). There were three long-term survivors with 57, 45, and 60 months of OS (#15, 14, 12); the last two of them are alive. Both OS and PFS were significantly lower in the presence of IMV-SV reconstruction (Figure 8c,d). The significance of other perioperative factors for the OS and PFS are shown in Figure 8e,f and Table S2. 

At the moment of the preparation of this manuscript, five patients are alive. Thirteen patients developed distant metastases, twice combined with local relapses. The most frequent first metastatic sites were the liver (n8) and peritoneum (n4). Two patients were relapse-free, with OS of 60 and 8 months (Table S1, #12,16). Portal vein thrombosis occurred in one patient 9 months after surgery, possibly caused by liquid restrictions, and it was the reason for the sole readmission. Ten months later, this patient died due to sepsis on the background of liver abscess perforation with no cancer relapse at surgery and autopsy (#9, Table S1). 

At the final pathological evaluation, mean tumor size was 35.8 ± 17.3 mm. There were no vein or perineural invasions in three cases with tumor regression grade Score ≤ 1. In two patients, a complete response after 12 courses of FOLFIRINOX was achieved (#15,16), but in case #15, distant metastases appeared 15 months after surgery.

Pretreatment and preoperative CA 19-9 levels were 357.9 [136; 532] and 34.3 [21; 44] U/l. The number of lymph nodes removed varied from 19 to 82 and was dependent on the extent of PE and lymphadenectomy. Nine patients had no regional lymph node invasion at pathological evaluation. Grade 2 tumors were met in ten cases. Gemcitabine- or FOLFIRINOX -based neoadjuvant chemotherapy was used in seven and nine patients, respectively, with both regimens used in four cases. The median number of neoadjuvant and adjuvant chemotherapy cycles was 6 [3; 9] (range 6–12) and 6 [4; 7] (range 4–28), respectively. Preoperative transaminase levels did not exceed two norms in all cases. 

### 3.4. T4 Group Results 

The T4 group included three patients after the R0-total PE accompanied by arterial resection and SMV excision with all its tributaries. In case #1, SMA resection was followed by IMV-SV anastomosis, and in two cases of SMA and common hepatic artery resection, there were no venous reconstructions. Demographic, clinical, and perioperative data of patients with T4 are presented in Table 3 and Table S1. At the final pathological evaluation, vein, arterial, and perineural invasion without significant tumor regression were documented in all cases. 

There were no postoperative deaths. One severe complication happened to patient #2—long-lasting (7 days) massive (up to 7 L a day) lymphorrhea—which we tried to treat by three unsuccessful attempts of performing mesocaval shunts, and which was gradually cured after the dilation of existing and/or development of additional collaterals. 

OS and PFS were 11, 23, and 18 months, and 11, 13, and 12 months, respectively. Patient #1 died of myocardial infarction having no relapse. 

## 4. Discussion

Superior mesenteric and/or portal vein resection with reconstruction is a standard approach for localized pancreatic cancer as comparable survival and was demonstrated after R0 PE with and without venous resection [26,27,28,29,30,31]. Resection of all except one first-order SMV branch could be performed if the remaining branch (resected and reconstructed or just preserved) assures collateral mesenteric drainage and liver supply [32], but an acute block of the PV and/or SMV can lead to mesenteric vein thrombosis and liver ischemia. If the PV/SMV route seems unreconstructable after resection during PE, today’s guidelines consider it locally advanced [9] and unresectable. In the case of slow-growing cancer and gradually developing SMV obstruction, collaterals are widening and bypassing the obstruction, preserving intestine-to-liver blood flow [33]. In some cases, due to chemotherapy and/or non-metastatic phenotype, cancer remains localized, and the conditions mentioned above give a chance for radical PE with SMV resection without PV-SMV reconstruction because of adequate collateral circulation. ISGPS elaborated on the classification system for extrahepatic porto-mesenteric venous resections, but vein resections without reconstruction of the SMV-PV route are not mentioned in this work [34].

The selection of patients for such extreme surgery cannot be overestimated. High-quality CT-based pancreatic protocol is the standard method for resectability assessment by the evaluation of vascular involvement and distant metastases with an accuracy of 77% [35,36]. MRI is an excellent modality for pancreatic cancer imaging and especially for liver metastases exclusion [37,38]. The likelihood of vascular invasion can be estimated based on the degree of circumferential tumor contact with the SMA, CHA, CT, PV-SMV, and the vessel’s wall irregularity, narrowing, or vascular occlusion [39,40]. The likelihood of vein invasion is up to 40%, 80%, and 100% with tumor contact ≤180°, >180°, and 360° correspondingly. CT predicts an involvement of veins in 40% of all cases [41,42] and is becoming unreliable for this purpose after neoadjuvant therapy [9,42].

We used different post-processing CT techniques for the assessment of the peripancreatic vessels and collateral venous blood flow adequacy, such as shaded surface display, maximum intensity projection, and 3D volume-rendered (VR) reconstruction imaging. Now, when planning SMV resection with excision of all its tributaries for “low” pancreatic cancer, we consider CT-based 3D-VR reconstruction the best and most necessary option for the depiction of the collateral venous system. Compared to the other rendering CT techniques, VR is superior in delineating vessels, pancreatic parenchyma, the tumor, and adjacent structures (Figure 1a–d, Figure 5a,b, Figure 6a,b and Figure 7a,b,d) [43,44,45].

To date, two relatively large (taking into consideration the rarity of the event) series of five [17] and six [46] PEs combined with SMV resection without reconstruction have been published. There was no mortality and severe morbidity in fifteen cases [14,15,16,17,18,47]. In eleven cases out of fifteen, authors dealt with ductal adenocarcinoma; in ten cases, SMV-SV confluence was preserved, IMV was the main collateral way from the intestine, and IMV was never transposed into the splenic vein. The work of Kulkarni et al. [36] is unusual because, except for two cases of PDAC, it described four SMV-PV resections without reconstruction in tumors with low malignant potential (neuroendocrine and solid-pseudopapillary neoplasms) and preservation of the distal SMV and right-sided collaterals in some cases. 

The low rate of severe morbidity and zero mortality in our series and in other works [14,15,16,17,18,46] suggest that compliance with selection criteria (see Methods) can make this surgery safe. We met severe complications (Dindo–Clavin 3b) in two patients, and in one of them, the massive and rapidly accumulated ascites was the reason for reoperation. This patient was the only one who did not have complete SMV occlusion (a filamentous gap remained). From this experience, we concluded that the complete block of the SMV lumen is one of the prerequisites for the safe execution of PE with the SMV resection with all its tributaries without PV-SMV reconstruction.

Clinically relevant pancreatic fistula (long-standing drain) developed in only one patient (8%) after twelve Whipple procedures, mainly because the hard pancreas and wide duct were met in 10 of these cases (Table S1).

The additional left SMV [47], twice detected on CT (Figure 1d) and confirmed at surgery, was not considered a reliable collateral, because of its much smaller diameter compared to occluded SMV and dilated IMV. 

The patients with combined arterial and vein involvement demonstrated the worst survival. At the same time, three patients of the T3 group (two of them alive) survived for 45, 57, and 60 months. In the last case, there is no relapse and the patient is a candidate for cure, still showing a non-metastatic phenotype despite the relatively small number of chemotherapy cycles. The survival after IMV-SV reconstruction was significantly lower compared to the cases without reconstruction (Figure 8c,d). We consider that reconstruction is not the real cause of that; being a reflection of the bigger tumor, total pancreatectomy (five of six cases), associated lower quality of life, and decreased chances for prolonged postoperative chemotherapy are viewed as the causes. The role of chemotherapy in the survival of these patients is underlined by the difference between median PFS and OS (Figure 8a,b) in the T3 group. 

The presented series demonstrates that: (1) under strictly definite conditions, the PE with SMV resection with all its tributaries without PV-SMV reconstruction for locally advanced pancreatic cancer can be performed safely; (2) resection of IMV followed by its anastomosis with splenic vein can be an option for such surgery; and (3) the long-term results of such procedures can be non-inferior to the results of the same procedures with the superior mesenteric vein resection and reconstruction [26,27,28,29,30,31]. 

The criteria for selection and the results of all the series on this subject can be helpful not only for surgeons, but also for tumor boards when a complex situation is under discussion. 

This series is the largest one dedicated to PE for ductal adenocarcinoma defined as locally advanced based on vein involvement criteria, and it gives an approximate explanation of the survival in this highly selected group. This strict selection, the small number of patients included, and the retrospective nature of the analysis of the procedures performed during quite a long period are the limitations of this study. We have no experience with left-sided PE with vein resection or any PE with SMV-SV confluence resection without PV-SMV reconstruction [15,46,48].

## 5. Conclusions 

The study of short- and long-term results of the relatively large series of PE with SMV resection without PV/SMV reconstruction for LA PDAC has shown zero mortality, acceptable morbidity, a relatively high level of radicality, and long-term results, non-inferior to the results of PE with the superior mesenteric vein resection and reconstruction. Strict selection based on rigorous mandatory criteria can make such surgery safe. CT-based preoperative reconstructions of the abdominal venous system are helpful instruments for the assessment of resectability, which cannot be underestimated. We hope that the data presented will be helpful for pancreatic surgeons dealing with LA PDAC.

## Figures and Tables

**Table 1:** Perioperative characteristics of the patients with SMV resection with all its tributaries without PV-SMV reconstruction, T3 group (n16).

Age, years	62 ± 8 (39–74)
Gender (f)	9 (56%)
Neoadjuvant chemotherapy	100%
Adjuvant chemotherapy (yes/no), n	15/1
GEM-based/FOLFIRINOX-mFOLFIRINOX, n	7/9
OP time (min)	401 ± 75 (195–475)
Estimated blood loss (mL)	346 ± 168 (100–550)
R0/R1, n (%)	13/3 (81%/19%)
IMV-SV reconstruction/No reconstruction, n (%)	5/11 (31%/69%)
Perineural invasion (yes/no)	14/2
Venous invasion at pathology (yes/no), n (%)	13/3 (81%/19%)
Tumor size, mm	36 ± 17 (0–48)
Number of lymph nodes removed	39 ± 17 (19–82)
Lymph nodes involvement pN0/pN1/pN2, n	9/3/4
Whipple/Total pancreatectomy, n	11/5

**Table 2:** Morbidity after SMV resection with all its tributaries without PV-SMV reconstruction, T3 group (n16).

Morbidity (C-D) 0-II/ ≥ III, n (%)	15/1 (94%/6%)
POPF No/ Grade A/B, n (%)	9/1/1 (82%/9%/9%)
Diarrhea, n (%)	2 (12,5%)
DGE Grade 1/2/3	3/3/1 (24%)
Hospital stay, days	13.6 ± 4 (7–23)
Lymphorrhea, n (%)	7 (44%)
Mortality, 90 days	0
Liver ischemia, n	0
Small bowel ischemia, n	0
Postop bleeding, n	0
Relaparotomy, n	1
Readmission, n (%)	1 (6%)
Bowel edema (2–3 days), n (%)	5 (31%)

Diarrhea—liquid stool > 4 times a day in a month after discharge. Lymphorrhea—drain lymph production of more than 500 mL a day in a month after surgery, C-D—Clavien-Dindo classification.

**Table 3:** Perioperative data and morbidity rate for pancreatectomies for T4 tumors (simultaneous arterial resection and SMV excision with all its tributaries).

Patient	1	2	3
Gender	f	m	m
BMI, kg/m^2^	19	21	21
Age, years	71	61	59
IMV-SV reconstruction	yes	no	no
CA 19-9, U/mL before NA/after NA	645/79	730/57	374/59
Neoadjuvant FOLFIRINOX, N of cycles	6	12	6
Adjuvant chemo, N of cycles	0	4	6
Histologic type, G	3	2	2
Lymph nodes removed, n	38	27	51
Lymph nodes involved, n	1	4	3
Tumor size at pathology, mm	38	27	51
Regression grade	3	3	2
Artery resected	SMA	SMA	CHA (Michels IX)
Blood transfusion (Units)	0	5	0
Morbidity, Dindo-C	0	3b	0
OP time, min	870	940	485
Blood loss, mL	450	1200	350
Complications	diarrhea	Lymphorrhea, bowel edema	
Readmission	yes	no	no
Length of stay, days	15	32	14
Reoperation	no	yes	no
Time of follow-up, months	12	24	20
OS	11	23	18
PFS	11	13	12
Relapse site	No, MI	liver	peritoneum

Diarrhea—liquid stool > 4 times a day in a month after discharge; lymphorrhea—drain production of more than 500 mL a day in a month after surgery; MI—myocardial infarction.

## Data Availability

The data supporting the reported results can be found in archived datasets of the Vishnevsky Institute of Surgery, the Bakhrushin Brothers Moscow City Hospital, and Ilyinskaya Hospital.

## Supplementary Materials

The following supporting information can be downloaded at: https://www.mdpi.com/article/10.3390/cancers16122234/s1, Table S1: Perioperative clinical and pathological data of the patients who underwent pancreatectomies with SMV resection without PV/SMV reconstruction for LA PDAC. Table S2: Statistical analysis of the short- and long-term results of the pancreatectomies with SMV resection without PV/SMV reconstruction for LA PDAC.
