# Supplementary material for: Pancreatectomy with En Bloc Superior Mesenteric Vein and All Its Tributaries Resection without PV/SMV Reconstruction for “Low” Locally Advanced Pancreatic Head Cancer
Source: Cancers (Basel). 2024 Jun 15;16(12):2234. doi: 10.3390/cancers16122234 (PMC11202096; doi:10.3390/cancers16122234)

Custom Tables

	Valid N	Mean	Standard Deviation	Median	Percentile 25	Percentile 75
BMI	16	21,9	1,8	22,0	20,5	23,5
Age	16	62,3	7,8	64,0	58,5	68,0
LN all	16	38,8	17,2	34,0	26,0	49,0
Tumor size, pathol, mm	16	35,8	17,3	38,5	27,5	47,0
OP time, min	16	400,9	75,4	395,0	325,0	460,0
Blood loss, ml	16	345,6	168,1	275,0	215,0	470,0
LOS, days	16	13,6	3,9	13,5	10,5	15,0
Time of follow-up	16	32	15	32	21	37

aplan-Meier

Case Processing Summary

Total N	N of Events	Censored	
		N	Percent
16	11	5	31,3%

Survival Table

	Time	Status	Cumulative Proportion Surviving at the Time		N of Cumulative Events	N of Remaining Cases
			Estimate	Std. Error		
1	8,000	no	.	.	0	15
2	12,000	yes	,933	,064	1	14
3	13,000	yes	,867	,088	2	13
4	19,000	yes	.	.	3	12
5	19,000	yes	,733	,114	4	11
6	21,000	yes	,667	,122	5	10
7	24,000	yes	,600	,126	6	9
8	25,000	yes	.	.	7	8
9	25,000	yes	,467	,129	8	7
10	27,000	yes	,400	,126	9	6
11	29,000	no	.	.	9	5
12	30,000	yes	,320	,124	10	4

13	30,000	no	.	.	10	3
14	45,000	no	.	.	10	2
15	57,000	yes	,160	,129	11	1
16	60,000	no	.	.	11	0

Means and Medians for Survival Time

Mean				Median			
		95% Confidence Interval				95% Confidence Interval	
Estimate	Std. Error	Lower Bound	Upper Bound	Estimate	Std. Error	Lower Bound	Upper Bound
33,453	4,682	24,276	42,631	25,000	1,932	21,213	28,787

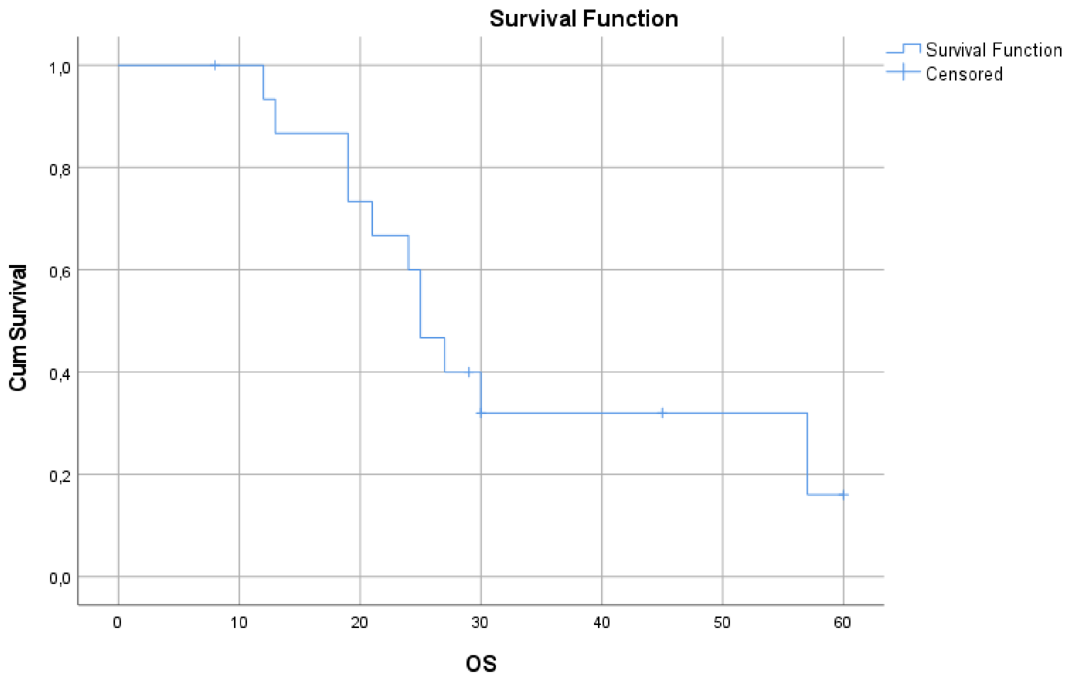

Kaplan-Meier

Kaplan-Meier

Case Processing Summary

NA Chemo >6	Total N	N of Events	Censored N	Censored Percent
no	12	9	3	25,0%
yes	4	1	3	75,0%
Overall	16	10	6	37,5%

Survival Table

NA Chemo >6		Time	Status	Cumulative Proportion Surviving at the Time Estimate	Cumulative Proportion Surviving at the Time Std. Error	N of Cumulative Events	N of Remaining Cases
no	1	12,000	yes	,917	,080	1	11
	2	13,000	yes	,833	,108	2	10
	3	19,000	yes	.	.	3	9
	4	19,000	yes	,667	,136	4	8
	5	21,000	yes	,583	,142	5	7
	6	24,000	yes	,500	,144	6	6
	7	25,000	yes	,417	,142	7	5
	8	27,000	yes	,333	,136	8	4
	9	29,000	no	.	.	8	3
	10	30,000	yes	,222	,128	9	2
	11	45,000	no	.	.	9	1
	12	60,000	no	.	.	9	0

yes	1	8,000	no	.	.	0	3
	2	25,000	yes	,667	,272	1	2
	3	30,000	no	.	.	1	1
	4	57,000	no	.	.	1	0

Means and Medians for Survival Time

	Mean				Median			
			95% Confidence Interval				95% Confidence Interval	
NA Chemo >6	Estimate	Std. Error	Lower Bound	Upper Bound	Estimate	Std. Error	Lower Bound	Upper Bound
no	30,000	5,050	20,102	39,898	24,000	3,464	17,210	30,790
yes	46,333	8,709	29,263	63,404	.	.	.	.
Overall	33,933	4,850	24,428	43,439	25,000	1,932	21,213	28,787

Overall Comparisons

	Chi-Square	df	Sig.
Log Rank (Mantel-Cox)	1,711	1	,191

Survival Functions

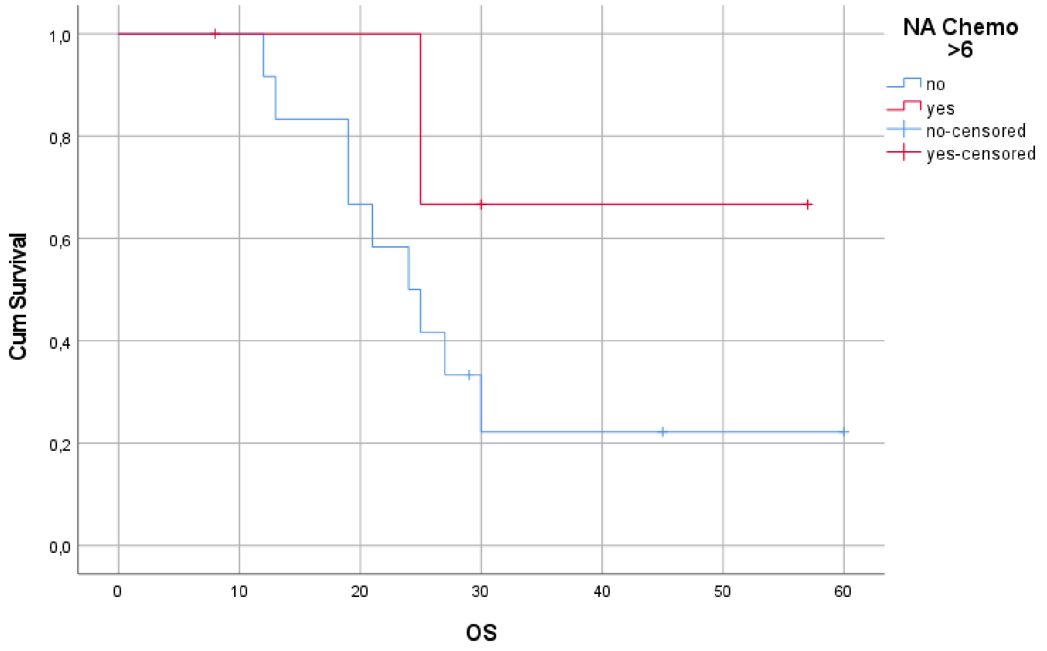

Kaplan-Meier

Case Processing Summary

LN all	Total N	N of Events	Censored	
			N	Percent
<= 40	10	6	4	40,0%
41+	5	3	2	40,0%
Overall	15	9	6	40,0%

Survival Table

LN all		Time	Status	Cumulative Proportion Surviving at the Time		N of Cumulative Events	N of Remaining Cases
				Estimate	Std. Error		
<= 40	1	8,000	no	.	.	0	9
	2	12,000	yes	,889	,105	1	8
	3	13,000	yes	,778	,139	2	7
	4	24,000	yes	,667	,157	3	6
	5	25,000	yes	.	.	4	5
	6	25,000	yes	,444	,166	5	4
	7	29,000	no	.	.	5	3
	8	30,000	yes	,296	,164	6	2
	9	57,000	no	.	.	6	1
	10	60,000	no	.	.	6	0
41+	1	19,000	yes	,800	,179	1	4
	2	21,000	yes	,600	,219	2	3
	3	27,000	yes	,400	,219	3	2
	4	30,000	no	.	.	3	1
	5	45,000	no	.	.	3	0

Means and Medians for Survival Time

LN all	Mean				Median			
	Estimate	Std. Error	95% Confidence Interval		Estimate	Std. Error	95% Confidence Interval	
			Lower Bound	Upper Bound			Lower Bound	Upper Bound
<= 40	33,222	6,356	20,765	45,680	25,000	,745	23,539	26,461
41+	31,400	5,104	21,397	41,403	27,000	6,573	14,118	39,882
Overall	35,000	5,077	25,048	44,952	25,000	1,871	21,333	28,667

Overall Comparisons

	Chi-Square	df	Sig.
Log Rank (Mantel-Cox)	,050	1	,824

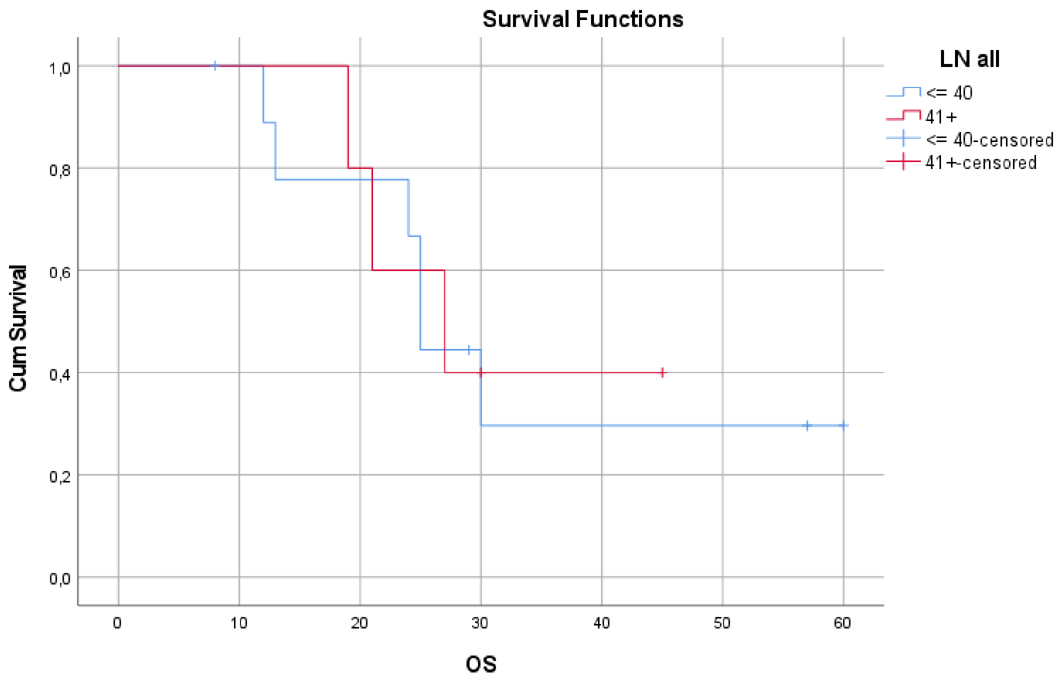

Kaplan-Meier

Case Processing Summary

LN, involved	Total N	N of Events	Censored N	Censored Percent
<= 0	9	4	5	55,6%
1+	7	6	1	14,3%
Overall	16	10	6	37,5%

Survival Table

LN, involved		Time	Status	Cumulative Proportion Surviving at the Time Estimate	Cumulative Proportion Surviving at the Time Std. Error	N of Cumulative Events	N of Remaining Cases
<= 0	1	8,000	no	.	.	0	8

	2	19,000	yes	,875	,117	1	7
	3	25,000	yes	,750	,153	2	6
	4	27,000	yes	,625	,171	3	5
	5	29,000	no	.	.	3	4
	6	30,000	yes	,469	,187	4	3
	7	30,000	no	.	.	4	2
	8	57,000	no	.	.	4	1
	9	60,000	no	.	.	4	0
1+	1	12,000	yes	,857	,132	1	6
	2	13,000	yes	,714	,171	2	5
	3	19,000	yes	,571	,187	3	4
	4	21,000	yes	,429	,187	4	3
	5	24,000	yes	,286	,171	5	2
	6	25,000	yes	,143	,132	6	1
	7	45,000	no	.	.	6	0

Means and Medians for Survival Time

	Mean				Median			
			95% Confidence Interval				95% Confidence Interval	
LN, involved	Estimate	Std. Error	Lower Bound	Upper Bound	Estimate	Std. Error	Lower Bound	Upper Bound
<= 0	41,688	6,433	29,080	54,295	30,000	.	.	.
1+	22,714	3,858	15,152	30,277	21,000	2,619	15,868	26,132
Overall	33,933	4,850	24,428	43,439	25,000	1,932	21,213	28,787

Overall Comparisons

	Chi-Square	df	Sig.
Log Rank (Mantel-Cox)	4,258	1	,039

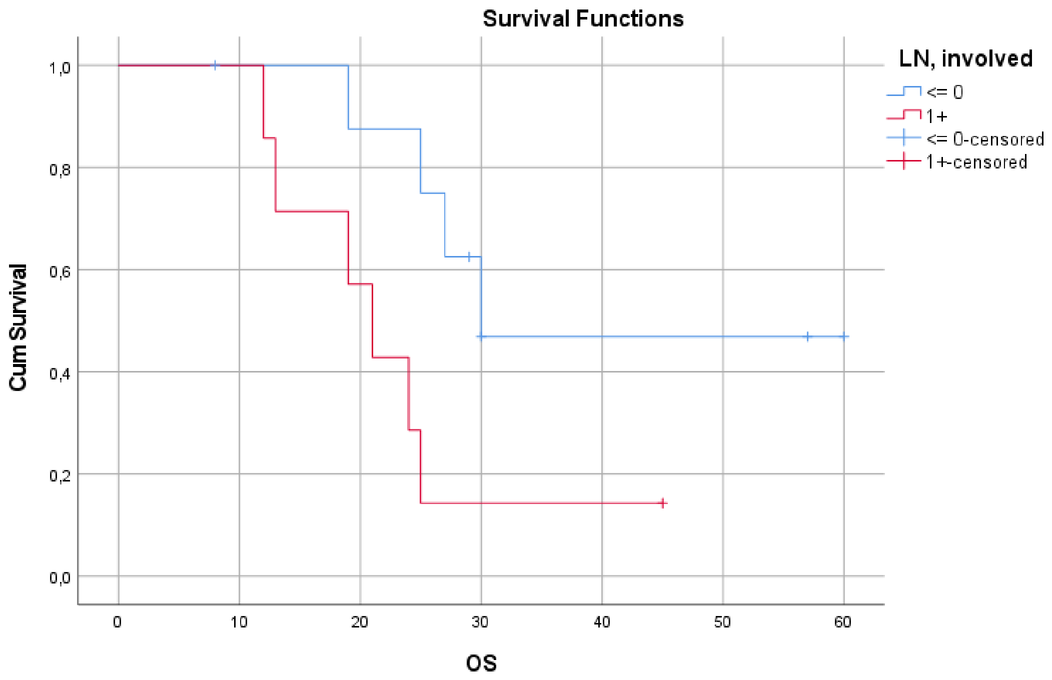

Kaplan-Meier

Case Processing Summary

Histologic type, G	Total N	N of Events	Censored	
			N	Percent
1 type	4	1	3	75,0%
2 or 3 type	12	9	3	25,0%
Overall	16	10	6	37,5%

Survival Table

Histologic type, G		Time	Status	Cumulative Proportion Surviving at the Time		N of Cumulative Events	N of Remaining Cases
				Estimate	Std. Error		
1 type	1	8,000	no	.	.	0	3
	2	25,000	yes	,667	,272	1	2
	3	45,000	no	.	.	1	1
	4	60,000	no	.	.	1	0
2 or 3 type	1	12,000	yes	,917	,080	1	11
	2	13,000	yes	,833	,108	2	10

3	19,000	yes	.	.	3	9
4	19,000	yes	,667	,136	4	8
5	21,000	yes	,583	,142	5	7
6	24,000	yes	,500	,144	6	6
7	25,000	yes	,417	,142	7	5
8	27,000	yes	,333	,136	8	4
9	29,000	no	.	.	8	3
10	30,000	yes	,222	,128	9	2
11	30,000	no	.	.	9	1
12	57,000	no	.	.	9	0

Means and Medians for Survival Time

	Mean				Median			
			95% Confidence Interval				95% Confidence Interval	
Histologic type, G	Estimate	Std. Error	Lower Bound	Upper Bound	Estimate	Std. Error	Lower Bound	Upper Bound
1 type	48,333	9,526	29,663	67,004	.	.	.	.
2 or 3 type	29,333	4,685	20,151	38,516	24,000	3,464	17,210	30,790
Overall	33,933	4,850	24,428	43,439	25,000	1,932	21,213	28,787

Overall Comparisons

	Chi-Square	df	Sig.
Log Rank (Mantel-Cox)	1,711	1	,191

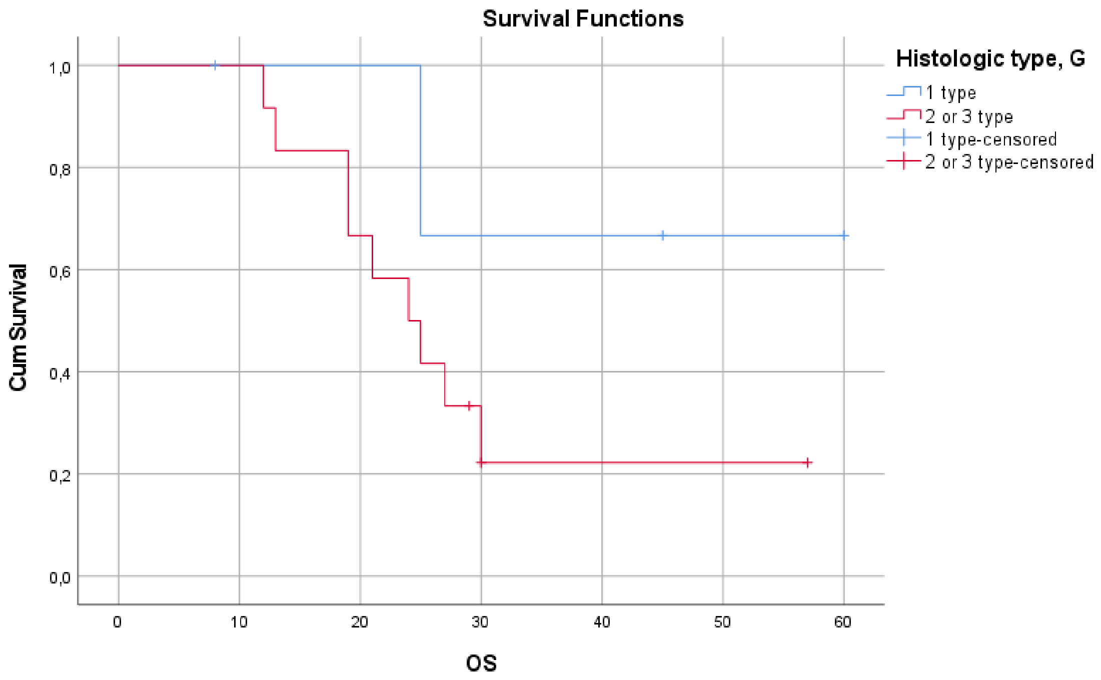

Kaplan-Meier

Warnings

No statistics are computed because all cases are censored.

Case Processing Summary

Regression grade	Total N	N of Events	Censored	
			N	Percent
0-1	3	0	3	100,0%
2+	13	10	3	23,1%
Overall	16	10	6	37,5%

Survival Table

Regression grade		Time	Status	Cumulative Proportion Surviving at the Time		N of Cumulative Events	N of Remaining Cases
				Estimate	Std. Error		
0-1	1	8,000	no	.	.	0	2

	2	57,000	no	.	.	0	1
2+	1	12,000	yes	,923	,074	1	12
	2	13,000	yes	,846	,100	2	11
	3	19,000	yes	.	.	3	10
	4	19,000	yes	,692	,128	4	9
	5	21,000	yes	,615	,135	5	8
	6	24,000	yes	,538	,138	6	7
	7	25,000	yes	.	.	7	6
	8	25,000	yes	,385	,135	8	5
	9	27,000	yes	,308	,128	9	4
	10	29,000	no	.	.	9	3
	11	30,000	yes	,205	,120	10	2
	12	30,000	no	.	.	10	1
	13	45,000	no	.	.	10	0

Overall Comparisons

	Chi-Square	df	Sig.
Log Rank (Mantel-Cox)	2,867	1	,090

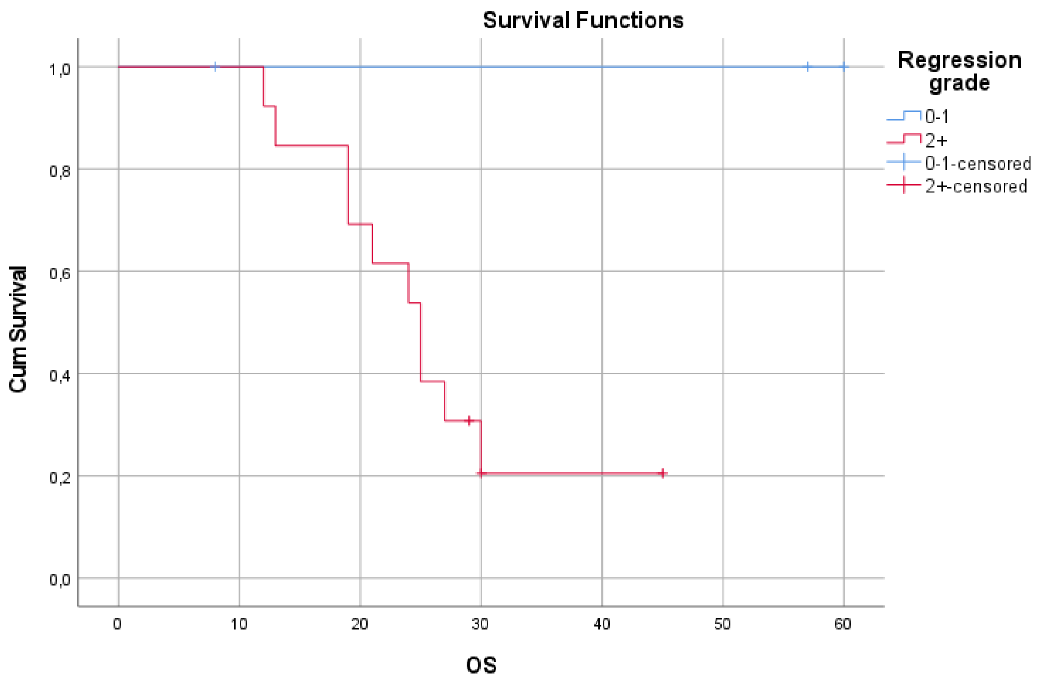

Kaplan-Meier

Case Processing Summary

Reconstruction	Total N	N of Events	Censored	
			N	Percent
no	11	5	6	54,5%
yes	5	5	0	0,0%
Overall	16	10	6	37,5%

Survival Table

Reconstruction		Time	Status	Cumulative Proportion Surviving at the Time		N of Cumulative Events	N of Remaining Cases
				Estimate	Std. Error		
no	1	8,000	no	.	.	0	10
	2	19,000	yes	,900	,095	1	9
	3	24,000	yes	,800	,126	2	8
	4	25,000	yes	.	.	3	7
	5	25,000	yes	,600	,155	4	6
	6	29,000	no	.	.	4	5
	7	30,000	yes	,480	,164	5	4
	8	30,000	no	.	.	5	3
	9	45,000	no	.	.	5	2
	10	57,000	no	.	.	5	1
	11	60,000	no	.	.	5	0
yes	1	12,000	yes	,800	,179	1	4
	2	13,000	yes	,600	,219	2	3
	3	19,000	yes	,400	,219	3	2
	4	21,000	yes	,200	,179	4	1
	5	27,000	yes	,000	,000	5	0

Means and Medians for Survival Time

Reconstruction	Mean				Median			
	Estimate	Std. Error	95% Confidence Interval		Estimate	Std. Error	95% Confidence Interval	
			Lower Bound	Upper Bound			Lower Bound	Upper Bound
no	41,700	5,771	30,388	53,012	30,000	.	.	.
yes	18,400	2,750	13,011	23,789	19,000	6,573	6,118	31,882
Overall	33,933	4,850	24,428	43,439	25,000	1,932	21,213	28,787

Overall Comparisons

	Chi-Square	df	Sig.
Log Rank (Mantel-Cox)	8,329	1	,004

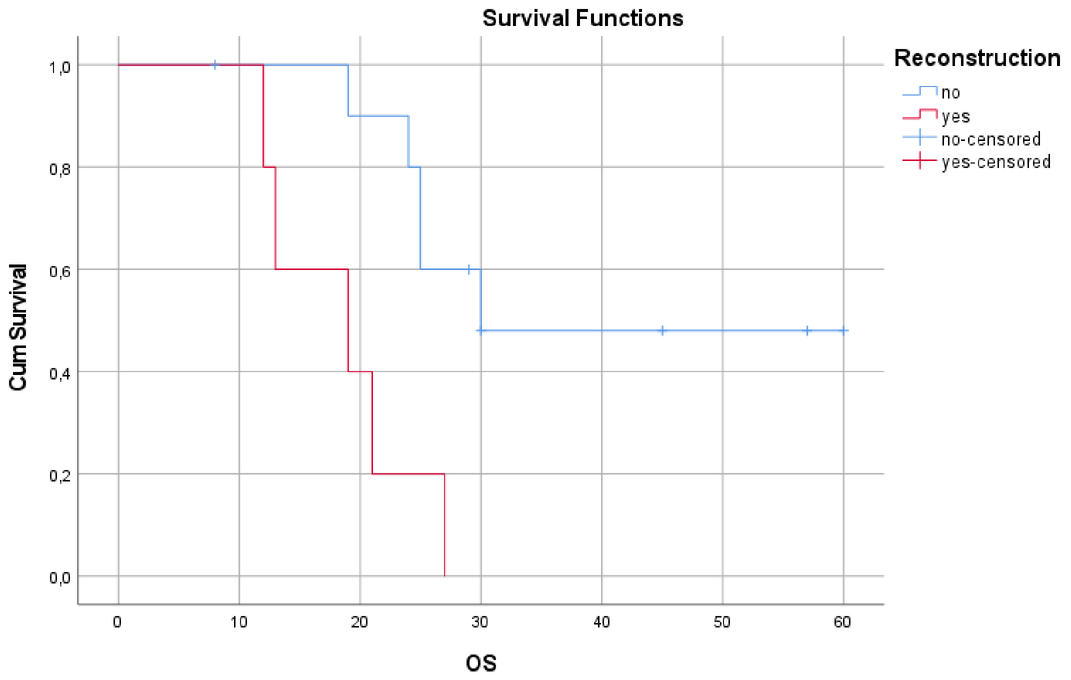

Kaplan-Meier

Case Processing Summary

R-status	Total N	N of Events	Censored	
			N	Percent
0	13	8	5	38,5%
1	3	2	1	33,3%
Overall	16	10	6	37,5%

Survival Table

R-status	Time	Status	Cumulative Proportion Surviving at the Time	N of Cumulative Events	N of Remaining Cases

				Estimate	Std. Error		
0	1	8,000	no	.	.	0	12
	2	13,000	yes	,917	,080	1	11
	3	19,000	yes	.	.	2	10
	4	19,000	yes	,750	,125	3	9
	5	21,000	yes	,667	,136	4	8
	6	24,000	yes	,583	,142	5	7
	7	25,000	yes	,500	,144	6	6
	8	27,000	yes	,417	,142	7	5
	9	29,000	no	.	.	7	4
	10	30,000	yes	,313	,140	8	3
	11	30,000	no	.	.	8	2
	12	57,000	no	.	.	8	1
	13	60,000	no	.	.	8	0
1	1	12,000	yes	,667	,272	1	2
	2	25,000	yes	,333	,272	2	1
	3	45,000	no	.	.	2	0

Means and Medians for Survival Time

	Mean				Median			
			95% Confidence Interval				95% Confidence Interval	
R-status	Estimate	Std. Error	Lower Bound	Upper Bound	Estimate	Std. Error	Lower Bound	Upper Bound
0	34,208	5,307	23,807	44,610	25,000	2,598	19,908	30,092
1	27,333	7,836	11,974	42,692	25,000	10,614	4,196	45,804
Overall	33,933	4,850	24,428	43,439	25,000	1,932	21,213	28,787

Overall Comparisons

	Chi-Square	df	Sig.
Log Rank (Mantel-Cox)	,023	1	,879

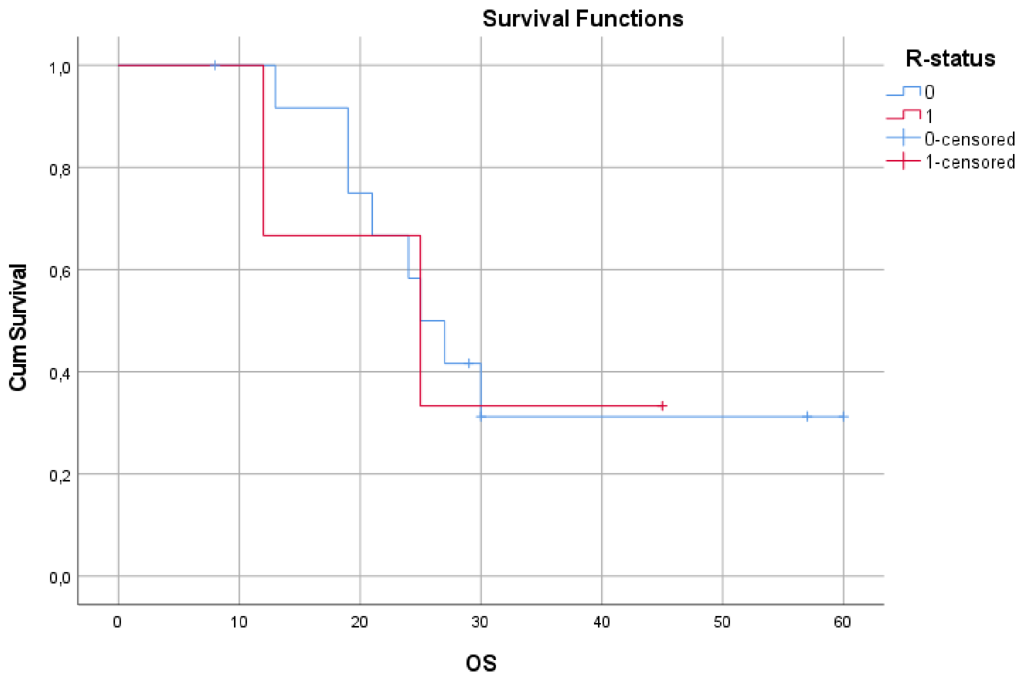

Kaplan-Meier

Case Processing Summary

CA 19-9 befor	Total N	N of Events	Censored N	Censored Percent
<= 319	8	5	3	37,5%
320+	7	4	3	42,9%
Overall	15	9	6	40,0%

Survival Table

CA 19-9 befor		Time	Status	Cumulative Proportion Surviving at the Time: Estimate	Cumulative Proportion Surviving at the Time: Std. Error	N of Cumulative Events	N of Remaining Cases
<= 319	1	12,000	yes	,875	,117	1	7
	2	13,000	yes	,750	,153	2	6
	3	25,000	yes	.	.	3	5
	4	25,000	yes	,500	,177	4	4
	5	27,000	yes	,375	,171	5	3
	6	29,000	no	.	.	5	2

	7	45,000	no	.	.	5	1
	8	60,000	no	.	.	5	0
320+	1	8,000	no	.	.	0	6
	2	19,000	yes	,833	,152	1	5
	3	21,000	yes	,667	,192	2	4
	4	24,000	yes	,500	,204	3	3
	5	30,000	yes	,333	,192	4	2
	6	30,000	no	.	.	4	1
	7	57,000	no	.	.	4	0

Means and Medians for Survival Time

	Mean				Median			
			95% Confidence Interval				95% Confidence Interval	
CA 19-9 befor	Estimate	Std. Error	Lower Bound	Upper Bound	Estimate	Std. Error	Lower Bound	Upper Bound
<= 319	35,250	7,017	21,496	49,004	25,000	6,600	12,065	37,935
320+	34,667	6,594	21,742	47,591	24,000	5,511	13,198	34,802
Overall	35,000	5,077	25,048	44,952	25,000	1,871	21,333	28,667

Overall Comparisons

	Chi-Square	df	Sig.
Log Rank (Mantel-Cox)	,002	1	,967

Survival Functions

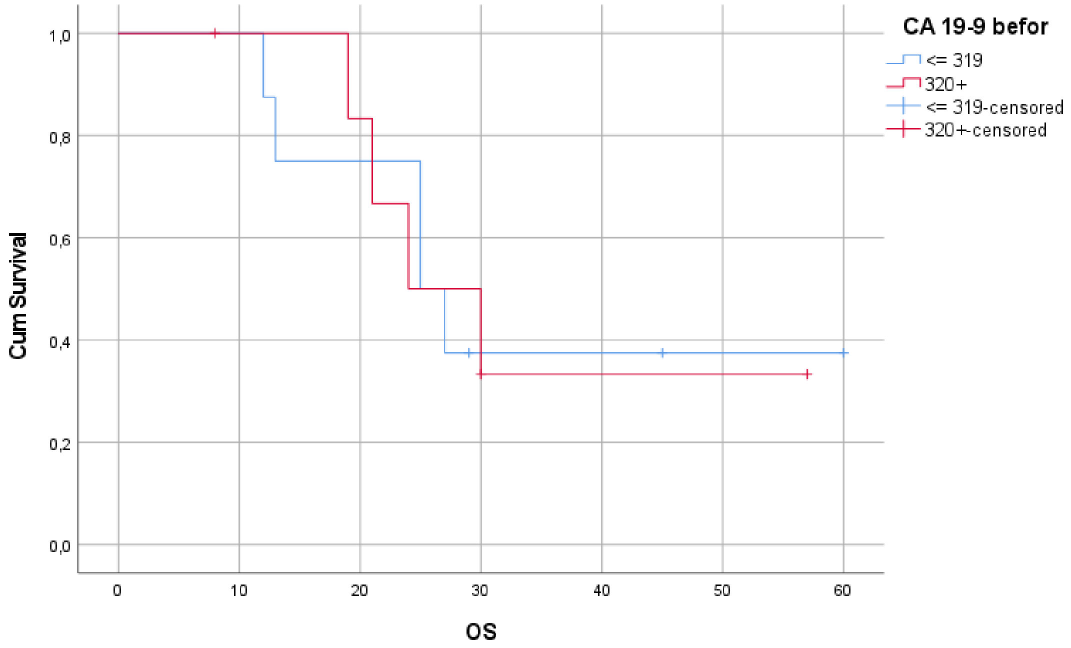

Kaplan-Meier

Case Processing Summary

CA 19-9 after/befor	Total N	N of Events	Censored	
			N	Percent
<= ,11	8	4	4	50,0%
,12+	7	5	2	28,6%
Overall	15	9	6	40,0%

Survival Table

CA 19-9 after/befor		Time	Status	Cumulative Proportion Surviving at the Time		N of Cumulative Events	N of Remaining Cases
				Estimate	Std. Error		
<= ,11	1	8,000	no	.	.	0	7
	2	19,000	yes	,857	,132	1	6
	3	24,000	yes	,714	,171	2	5
	4	25,000	yes	,571	,187	3	4
	5	29,000	no	.	.	3	3
	6	30,000	yes	,381	,199	4	2
	7	30,000	no	.	.	4	1
	8	57,000	no	.	.	4	0
,12+	1	12,000	yes	,857	,132	1	6
	2	13,000	yes	,714	,171	2	5
	3	21,000	yes	,571	,187	3	4
	4	25,000	yes	,429	,187	4	3
	5	27,000	yes	,286	,171	5	2
	6	45,000	no	.	.	5	1
	7	60,000	no	.	.	5	0

Means and Medians for Survival Time

CA 19-9 after/befor	Mean				Median			
			95% Confidence Interval				95% Confidence Interval	
	Estimate	Std. Error	Lower Bound	Upper Bound	Estimate	Std. Error	Lower Bound	Upper Bound

<= ,11	37,143	6,365	24,668	49,617	30,000	5,233	19,744	40,256
,12+	31,143	7,170	17,090	45,196	25,000	5,237	14,735	35,265
Overall	35,000	5,077	25,048	44,952	25,000	1,871	21,333	28,667

Overall Comparisons

	Chi-Square	df	Sig.
Log Rank (Mantel-Cox)	,513	1	,474

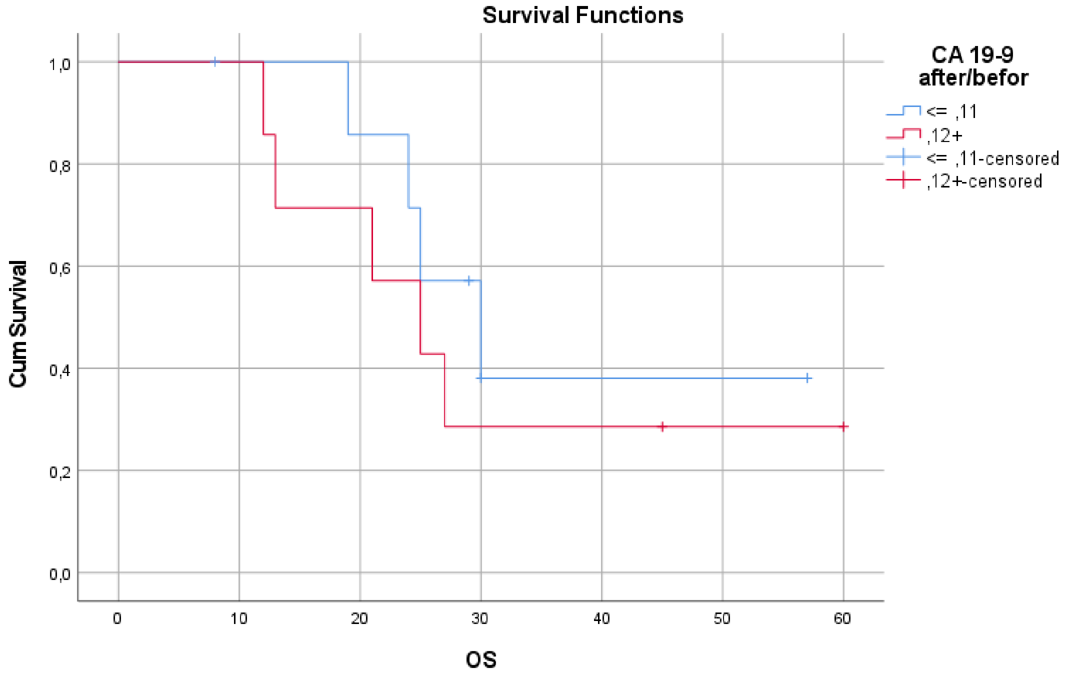

Kaplan-Meier

Case Processing Summary

Total N	N of Events	Censored N	Censored Percent
16	13	3	18,8%

Survival Table

	Time	Status	Cumulative Proportion Surviving at the Time		N of Cumulative Events	N of Remaining Cases
			Estimate	Std. Error		
1	6,000	yes	,938	,061	1	15
2	8,000	no	.	.	1	14
3	11,000	yes	.	.	2	13
4	11,000	yes	,804	,102	3	12
5	14,000	yes	,737	,113	4	11
6	15,000	yes	,670	,121	5	10
7	16,000	yes	,603	,126	6	9
8	18,000	yes	.	.	7	8
9	18,000	yes	,469	,129	8	7
10	19,000	yes	.	.	9	6
11	19,000	yes	,335	,122	10	5
12	21,000	yes	.	.	11	4
13	21,000	yes	,201	,104	12	3
14	25,000	no	.	.	12	2
15	29,000	yes	,100	,088	13	1
16	60,000	no	.	.	13	0

Means and Medians for Survival Time

Mean				Median			
		95% Confidence Interval				95% Confidence Interval	
Estimate	Std. Error	Lower Bound	Upper Bound	Estimate	Std. Error	Lower Bound	Upper Bound
21,569	3,825	14,072	29,066	18,000	1,443	15,171	20,829

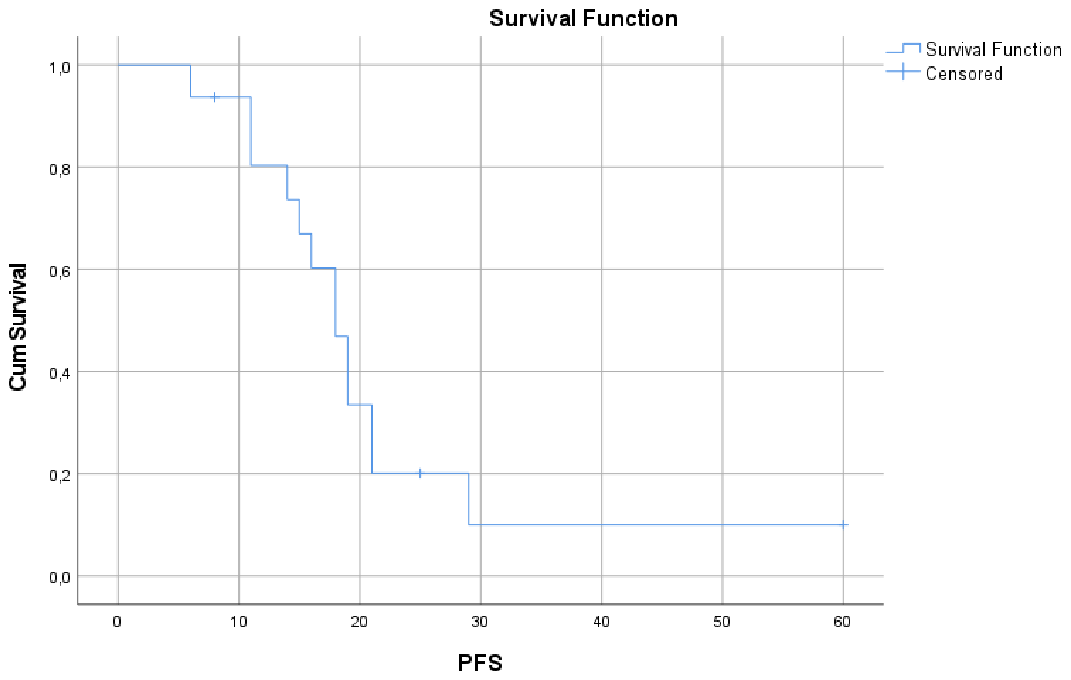

Kaplan-Meier

Case Processing Summary

NA Chemo >6	Total N	N of Events	Censored	
			N	Percent
no	12	11	1	8,3%
yes	4	2	2	50,0%
Overall	16	13	3	18,8%

Survival Table

NA Chemo >6		Time	Status	Cumulative Proportion Surviving at the Time		N of Cumulative Events	N of Remaining Cases
				Estimate	Std. Error		
no	1	6,000	yes	,917	,080	1	11
	2	11,000	yes	.	.	2	10
	3	11,000	yes	,750	,125	3	9
	4	15,000	yes	,667	,136	4	8
	5	16,000	yes	,583	,142	5	7
	6	18,000	yes	.	.	6	6
	7	18,000	yes	,417	,142	7	5

	8	19,000	yes	.	.	8	4
	9	19,000	yes	,250	,125	9	3
	10	21,000	yes	,167	,108	10	2
	11	29,000	yes	,083	,080	11	1
	12	60,000	no	.	.	11	0
yes	1	8,000	no	.	.	0	3
	2	14,000	yes	,667	,272	1	2
	3	21,000	yes	,333	,272	2	1
	4	25,000	no	.	.	2	0

Means and Medians for Survival Time

NA Chemo >6	Mean				Median			
	Estimate	Std. Error	95% Confidence Interval		Estimate	Std. Error	95% Confidence Interval	
			Lower Bound	Upper Bound			Lower Bound	Upper Bound
no	20,250	3,810	12,783	27,717	18,000	1,708	14,653	21,347
yes	20,000	2,625	14,856	25,144	21,000	5,715	9,798	32,202
Overall	21,569	3,825	14,072	29,066	18,000	1,443	15,171	20,829

Overall Comparisons

	Chi-Square	df	Sig.
Log Rank (Mantel-Cox)	,635	1	,425

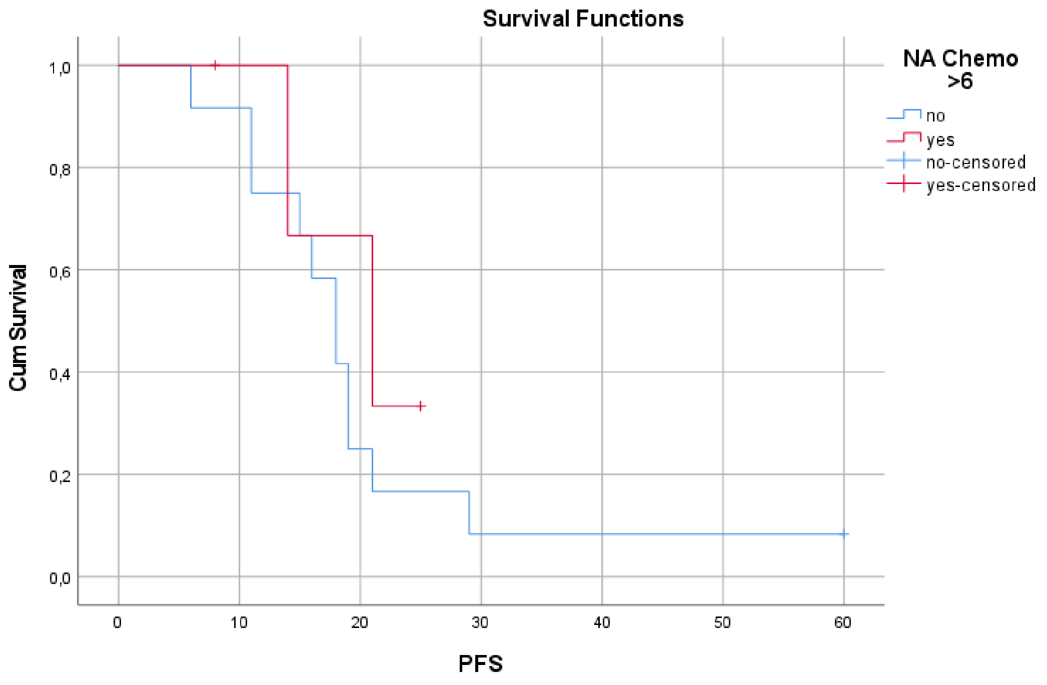

Kaplan-Meier

Case Processing Summary

LN all	Total N	N of Events	Censored N	Censored Percent
<= 40	10	7	3	30,0%
41+	5	5	0	0,0%
Overall	15	12	3	20,0%

Survival Table

LN all		Time	Status	Cumulative Proportion Surviving at the Time: Estimate	Cumulative Proportion Surviving at the Time: Std. Error	N of Cumulative Events	N of Remaining Cases
<= 40	1	6,000	yes	,900	,095	1	9
	2	8,000	no	.	.	1	8
	3	11,000	yes	,788	,134	2	7
	4	18,000	yes	,675	,155	3	6
	5	19,000	yes	.	.	4	5
	6	19,000	yes	,450	,166	5	4
	7	21,000	yes	.	.	6	3
	8	21,000	yes	,225	,140	7	2
	9	25,000	no	.	.	7	1
	10	60,000	no	.	.	7	0
41+	1	11,000	yes	,800	,179	1	4
	2	14,000	yes	,600	,219	2	3
	3	15,000	yes	,400	,219	3	2
	4	18,000	yes	,200	,179	4	1
	5	29,000	yes	,000	,000	5	0

Means and Medians for Survival Time

	Mean				Median			
			95% Confidence Interval				95% Confidence Interval	
LN all	Estimate	Std. Error	Lower Bound	Upper Bound	Estimate	Std. Error	Lower Bound	Upper Bound

<= 40	26,363	6,221	14,169	38,556	19,000	,738	17,554	20,446
41+	17,400	3,108	11,308	23,492	15,000	1,095	12,853	17,147
Overall	21,974	4,078	13,981	29,967	19,000	1,788	15,496	22,504

Overall Comparisons

	Chi-Square	df	Sig.
Log Rank (Mantel-Cox)	1,228	1	,268

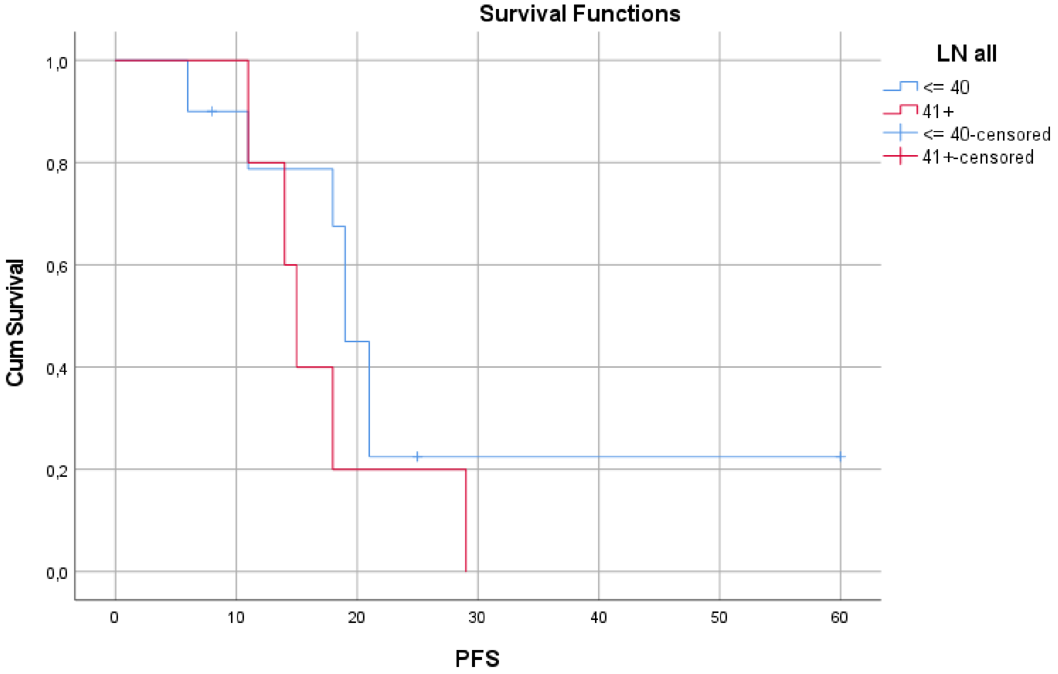

Kaplan-Meier

Case Processing Summary

LN, involved	Total N	N of Events	Censored	
			N	Percent
<= 0	9	7	2	22,2%
1+	7	6	1	14,3%
Overall	16	13	3	18,8%

Survival Table

LN, involved		Time	Status	Cumulative Proportion Surviving at the Time: Estimate	Std. Error	N of Cumulative Events	N of Remaining Cases
<= 0	1	8,000	no	.	.	0	8
	2	11,000	yes	,875	,117	1	7
	3	14,000	yes	,750	,153	2	6
	4	18,000	yes	.	.	3	5
	5	18,000	yes	,500	,177	4	4
	6	19,000	yes	,375	,171	5	3
	7	21,000	yes	.	.	6	2
	8	21,000	yes	,125	,117	7	1
	9	60,000	no	.	.	7	0
1+	1	6,000	yes	,857	,132	1	6
	2	11,000	yes	,714	,171	2	5
	3	15,000	yes	,571	,187	3	4
	4	16,000	yes	,429	,187	4	3
	5	19,000	yes	,286	,171	5	2
	6	25,000	no	.	.	5	1
	7	29,000	yes	,000	,000	6	0

Means and Medians for Survival Time

LN, involved	Mean: Estimate	Mean: Std. Error	Mean: 95% Confidence Interval Lower Bound	Mean: 95% Confidence Interval Upper Bound	Median: Estimate	Median: Std. Error	Median: 95% Confidence Interval Lower Bound	Median: 95% Confidence Interval Upper Bound
<= 0	22,750	5,104	12,745	32,755	18,000	2,357	13,380	22,620
1+	17,857	3,315	11,359	24,355	16,000	1,309	13,434	18,566
Overall	21,569	3,825	14,072	29,066	18,000	1,443	15,171	20,829

Overall Comparisons

	Chi-Square	df	Sig.
Log Rank (Mantel-Cox)	,131	1	,717

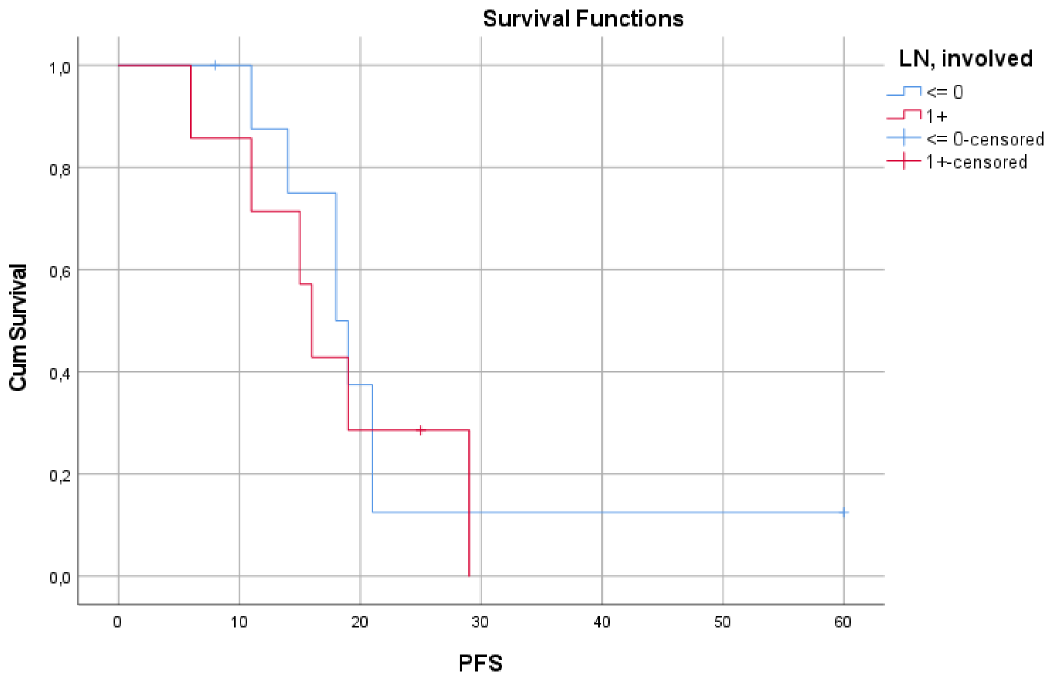

Kaplan-Meier

Case Processing Summary

Histologic type, G	Total N	N of Events	Censored N	Censored Percent
1 type	4	1	3	75,0%
2 or 3 type	12	12	0	0,0%
Overall	16	13	3	18,8%

Survival Table

Histologic type, G		Time	Status	Cumulative Proportion Surviving at the Time Estimate	Cumulative Proportion Surviving at the Time Std. Error	N of Cumulative Events	N of Remaining Cases
1 type	1	8,000	no	.	.	0	3
	2	25,000	no	.	.	0	2
	3	29,000	yes	,500	,354	1	1
	4	60,000	no	.	.	1	0
2 or 3 type	1	6,000	yes	,917	,080	1	11
	2	11,000	yes	.	.	2	10

3	11,000	yes	,750	,125	3	9
4	14,000	yes	,667	,136	4	8
5	15,000	yes	,583	,142	5	7
6	16,000	yes	,500	,144	6	6
7	18,000	yes	.	.	7	5
8	18,000	yes	,333	,136	8	4
9	19,000	yes	.	.	9	3
10	19,000	yes	,167	,108	10	2
11	21,000	yes	.	.	11	1
12	21,000	yes	,000	,000	12	0

Means and Medians for Survival Time

	Mean				Median			
			95% Confidence Interval				95% Confidence Interval	
Histologic type, G	Estimate	Std. Error	Lower Bound	Upper Bound	Estimate	Std. Error	Lower Bound	Upper Bound
1 type	44,500	10,960	23,018	65,982	29,000	.	.	.
2 or 3 type	15,750	1,321	13,161	18,339	16,000	1,732	12,605	19,395
Overall	21,569	3,825	14,072	29,066	18,000	1,443	15,171	20,829

Overall Comparisons

	Chi-Square	df	Sig.
Log Rank (Mantel-Cox)	7,930	1	,005

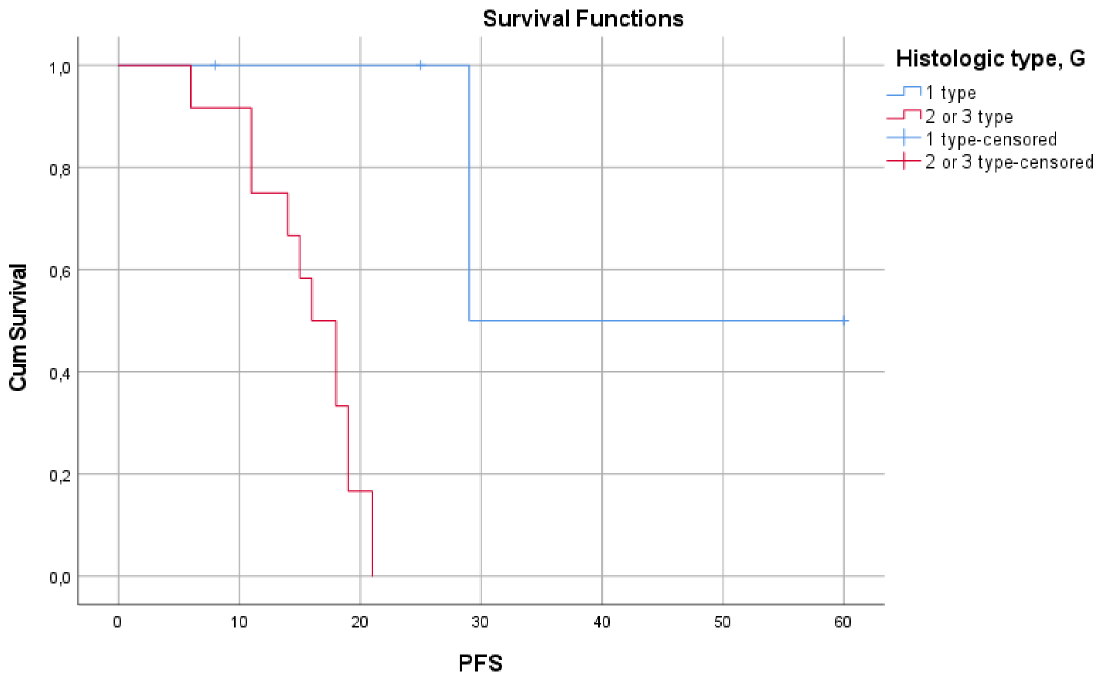

Kaplan-Meier

Case Processing Summary

Regression grade	Total N	N of Events	Censored N	Censored Percent
0-1	3	1	2	66,7%
2+	13	12	1	7,7%
Overall	16	13	3	18,8%

Survival Table

Regression grade		Time	Status	Cumulative Proportion Surviving at the Time: Estimate	Cumulative Proportion Surviving at the Time: Std. Error	N of Cumulative Events	N of Remaining Cases
0-1	1	8,000	no	.	.	0	2
	2	21,000	yes	,500	,354	1	1
	3	60,000	no	.	.	1	0
2+	1	6,000	yes	,923	,074	1	12
	2	11,000	yes	.	.	2	11
	3	11,000	yes	,769	,117	3	10

4	14,000	yes	,692	,128	4	9
5	15,000	yes	,615	,135	5	8
6	16,000	yes	,538	,138	6	7
7	18,000	yes	.	.	7	6
8	18,000	yes	,385	,135	8	5
9	19,000	yes	.	.	9	4
10	19,000	yes	,231	,117	10	3
11	21,000	yes	,154	,100	11	2
12	25,000	no	.	.	11	1
13	29,000	yes	,000	,000	12	0

Means and Medians for Survival Time

	Mean				Median			
			95% Confidence Interval				95% Confidence Interval	
Regression grade	Estimate	Std. Error	Lower Bound	Upper Bound	Estimate	Std. Error	Lower Bound	Upper Bound
0-1	40,500	13,789	13,474	67,526	21,000	.	.	.
2+	17,385	1,830	13,797	20,972	18,000	1,754	14,562	21,438
Overall	21,569	3,825	14,072	29,066	18,000	1,443	15,171	20,829

Overall Comparisons

	Chi-Square	df	Sig.
Log Rank (Mantel-Cox)	2,629	1	,105

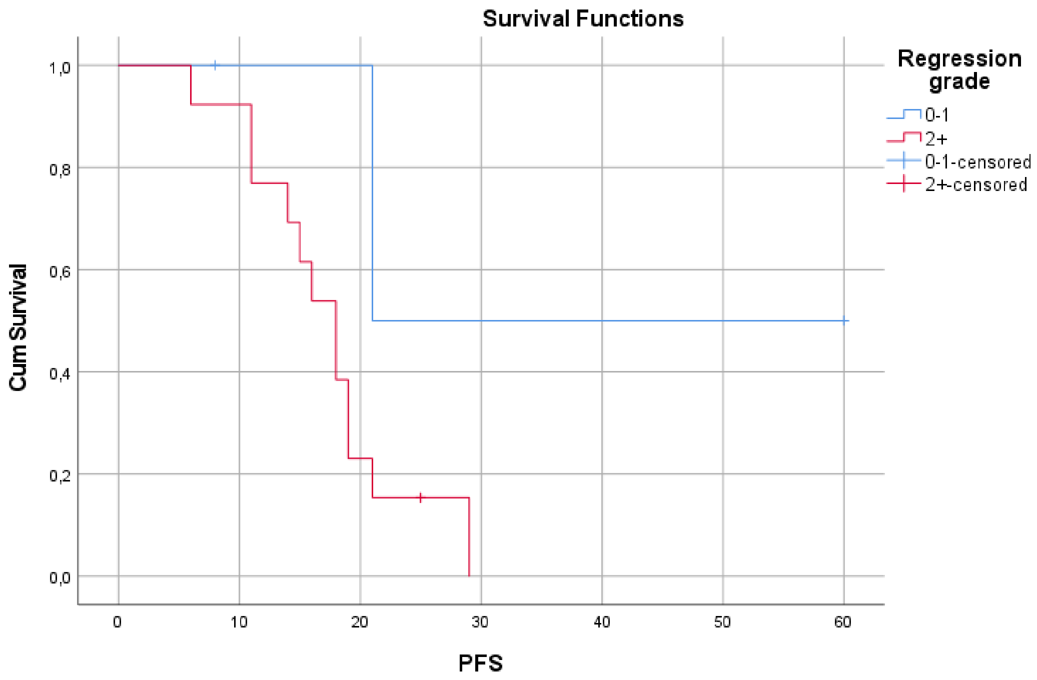

Kaplan-Meier

Case Processing Summary

Reconstruction	Total N	N of Events	Censored	
			N	Percent
no	11	8	3	27,3%
yes	5	5	0	0,0%
Overall	16	13	3	18,8%

Survival Table

Reconstruction		Time	Status	Cumulative Proportion Surviving at the Time		N of Cumulative Events	N of Remaining Cases
				Estimate	Std. Error		
no	1	8,000	no	.	.	0	10
	2	14,000	yes	,900	,095	1	9
	3	16,000	yes	,800	,126	2	8
	4	18,000	yes	,700	,145	3	7
	5	19,000	yes	.	.	4	6
	6	19,000	yes	,500	,158	5	5

	7	21,000	yes	.	.	6	4
	8	21,000	yes	,300	,145	7	3
	9	25,000	no	.	.	7	2
	10	29,000	yes	,150	,128	8	1
	11	60,000	no	.	.	8	0
yes	1	6,000	yes	,800	,179	1	4
	2	11,000	yes	.	.	2	3
	3	11,000	yes	,400	,219	3	2
	4	15,000	yes	,200	,179	4	1
	5	18,000	yes	,000	,000	5	0

Means and Medians for Survival Time

	Mean				Median			
			95% Confidence Interval				95% Confidence Interval	
Reconstruction	Estimate	Std. Error	Lower Bound	Upper Bound	Estimate	Std. Error	Lower Bound	Upper Bound
no	26,150	5,063	16,226	36,074	19,000	1,186	16,676	21,324
yes	12,200	2,035	8,212	16,188	11,000	2,739	5,632	16,368
Overall	21,569	3,825	14,072	29,066	18,000	1,443	15,171	20,829

Overall Comparisons

	Chi-Square	df	Sig.
Log Rank (Mantel-Cox)	10,394	1	,001

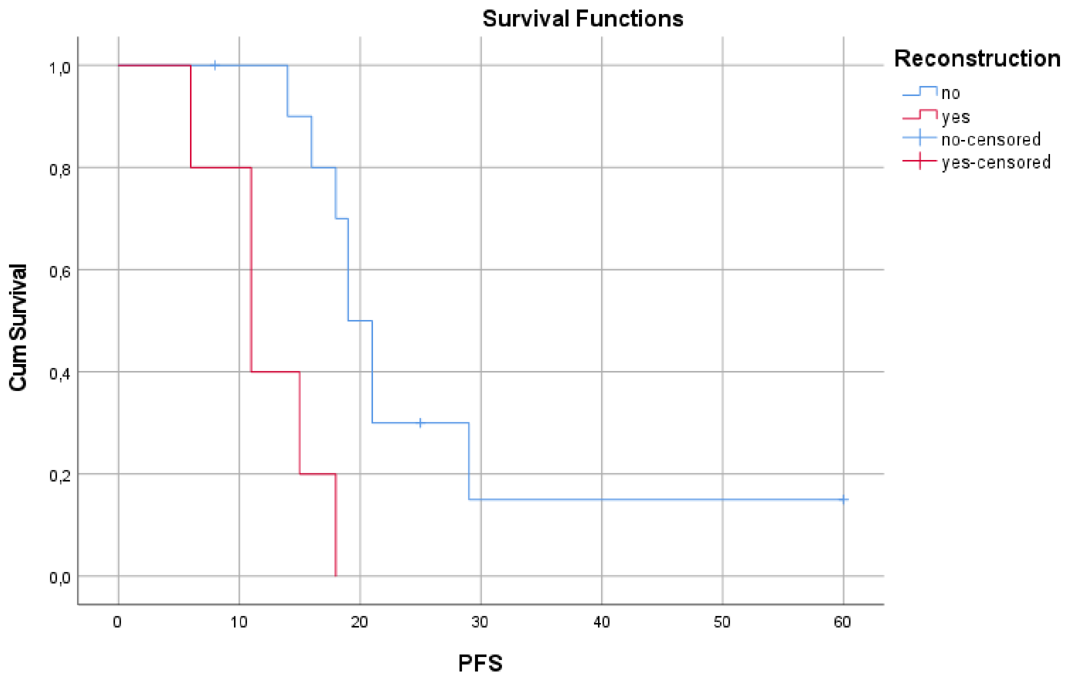

Kaplan-Meier

Case Processing Summary

R-status	Total N	N of Events	Censored N	Censored Percent
0	13	11	2	15,4%
1	3	2	1	33,3%
Overall	16	13	3	18,8%

Survival Table

R-status		Time	Status	Cumulative Proportion Surviving at the Time Estimate	Cumulative Proportion Surviving at the Time Std. Error	N of Cumulative Events	N of Remaining Cases
0	1	8,000	no	.	.	0	12
	2	11,000	yes	.	.	1	11
	3	11,000	yes	,833	,108	2	10
	4	14,000	yes	,750	,125	3	9
	5	15,000	yes	,667	,136	4	8
	6	16,000	yes	,583	,142	5	7

	7	18,000	yes	.	.	6	6
	8	18,000	yes	,417	,142	7	5
	9	19,000	yes	.	.	8	4
	10	19,000	yes	,250	,125	9	3
	11	21,000	yes	.	.	10	2
	12	21,000	yes	,083	,080	11	1
	13	60,000	no	.	.	11	0
1	1	6,000	yes	,667	,272	1	2
	2	25,000	no	.	.	1	1
	3	29,000	yes	,000	,000	2	0

Means and Medians for Survival Time

	Mean				Median			
			95% Confidence Interval				95% Confidence Interval	
R-status	Estimate	Std. Error	Lower Bound	Upper Bound	Estimate	Std. Error	Lower Bound	Upper Bound
0	20,250	3,585	13,224	27,276	18,000	1,708	14,653	21,347
1	21,333	8,853	3,982	38,685	29,000	,000	.	.
Overall	21,569	3,825	14,072	29,066	18,000	1,443	15,171	20,829

Overall Comparisons

	Chi-Square	df	Sig.
Log Rank (Mantel-Cox)	,861	1	,354

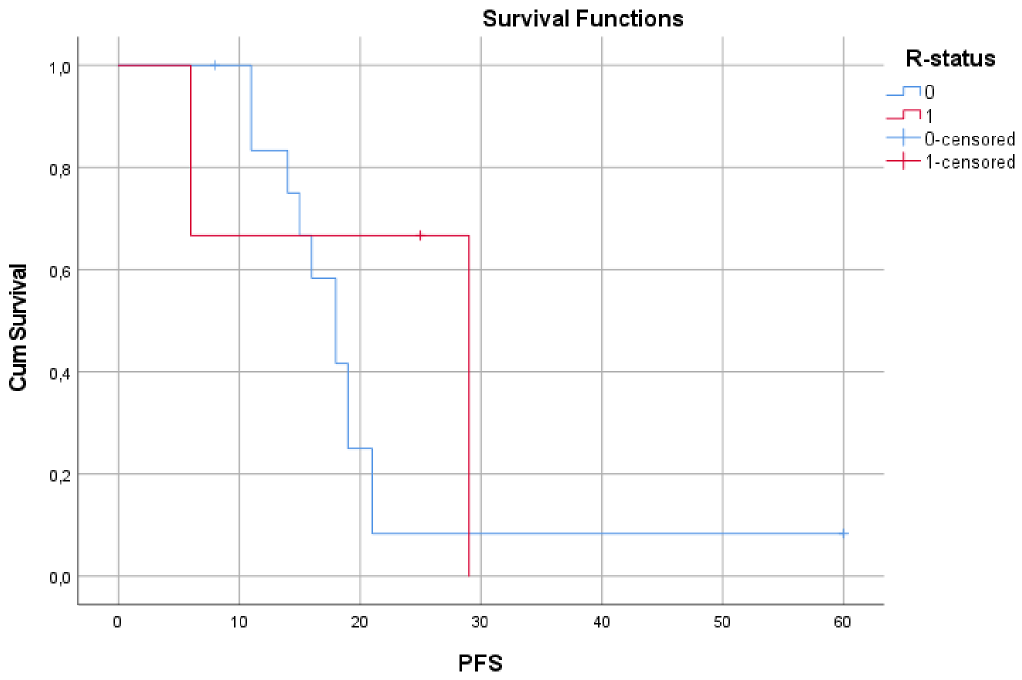

Kaplan-Meier

Case Processing Summary

CA 19-9 befor	Total N	N of Events	Censored N	Censored Percent
<= 319	8	6	2	25,0%
320+	7	6	1	14,3%
Overall	15	12	3	20,0%

Survival Table

CA 19-9 befor		Time	Status	Cumulative Proportion Surviving at the Time Estimate	Cumulative Proportion Surviving at the Time Std. Error	N of Cumulative Events	N of Remaining Cases
<= 319	1	6,000	yes	,875	,117	1	7
	2	11,000	yes	,750	,153	2	6
	3	18,000	yes	.	.	3	5
	4	18,000	yes	,500	,177	4	4
	5	19,000	yes	,375	,171	5	3
	6	25,000	no	.	.	5	2

	7	29,000	yes	,188	,158	6	1
	8	60,000	no	.	.	6	0
320+	1	8,000	no	.	.	0	6
	2	11,000	yes	,833	,152	1	5
	3	14,000	yes	,667	,192	2	4
	4	15,000	yes	,500	,204	3	3
	5	19,000	yes	,333	,192	4	2
	6	21,000	yes	.	.	5	1
	7	21,000	yes	,000	,000	6	0

Means and Medians for Survival Time

	Mean				Median			
			95% Confidence Interval				95% Confidence Interval	
CA 19-9 befor	Estimate	Std. Error	Lower Bound	Upper Bound	Estimate	Std. Error	Lower Bound	Upper Bound
<= 319	25,688	6,742	12,473	38,902	18,000	3,771	10,608	25,392
320+	16,833	1,682	13,537	20,129	15,000	3,062	8,999	21,001
Overall	21,974	4,078	13,981	29,967	19,000	1,788	15,496	22,504

Overall Comparisons

	Chi-Square	df	Sig.
Log Rank (Mantel-Cox)	,873	1	,350

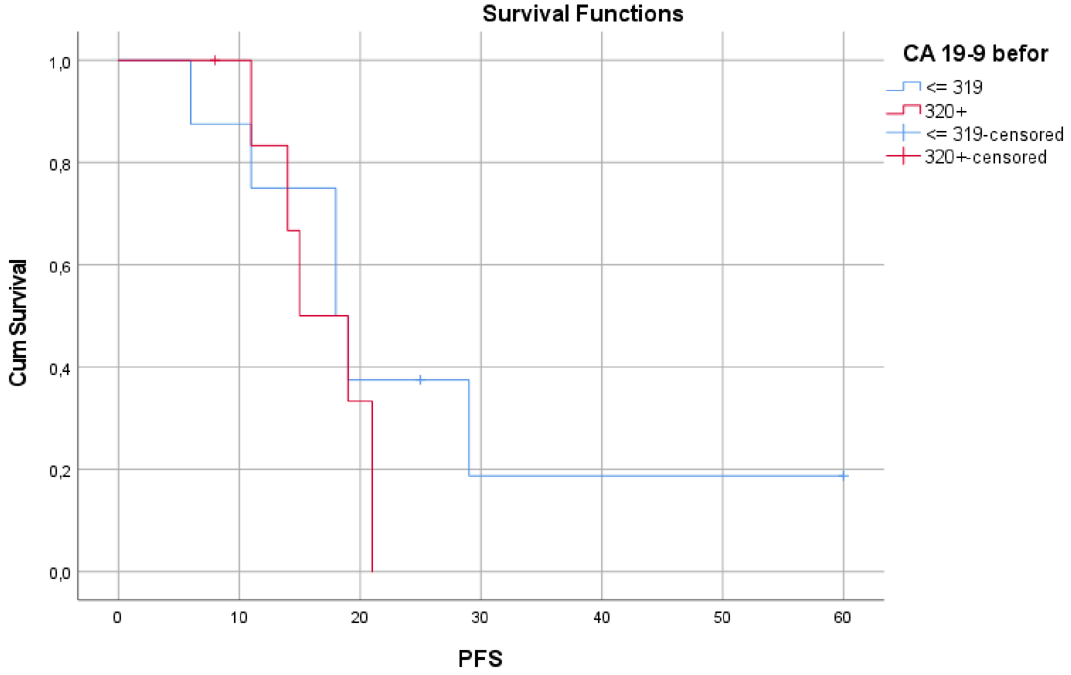

Kaplan-Meier

Case Processing Summary

CA 19-9 after/befor	Total N	N of Events	Censored	
			N	Percent
<= ,11	8	6	2	25,0%
,12+	7	6	1	14,3%
Overall	15	12	3	20,0%

Survival Table

CA 19-9 after/befor		Time	Status	Cumulative Proportion Surviving at the Time		N of Cumulative Events	N of Remaining Cases
				Estimate	Std. Error		
<= ,11	1	8,000	no	.	.	0	7
	2	11,000	yes	,857	,132	1	6
	3	14,000	yes	,714	,171	2	5
	4	19,000	yes	.	.	3	4
	5	19,000	yes	,429	,187	4	3
	6	21,000	yes	.	.	5	2
	7	21,000	yes	,143	,132	6	1
	8	25,000	no	.	.	6	0
,12+	1	6,000	yes	,857	,132	1	6
	2	11,000	yes	,714	,171	2	5
	3	15,000	yes	,571	,187	3	4
	4	18,000	yes	.	.	4	3
	5	18,000	yes	,286	,171	5	2
	6	29,000	yes	,143	,132	6	1
	7	60,000	no	.	.	6	0

Means and Medians for Survival Time

CA 19-9 after/befor	Mean				Median			
	Estimate	Std. Error	95% Confidence Interval		Estimate	Std. Error	95% Confidence Interval	
			Lower Bound	Upper Bound			Lower Bound	Upper Bound

<= ,11	18,571	1,640	15,358	21,785	19,000	3,273	12,584	25,416
,12+	22,429	6,308	10,065	34,792	18,000	1,793	14,486	21,514
Overall	21,974	4,078	13,981	29,967	19,000	1,788	15,496	22,504

Overall Comparisons

	Chi-Square	df	Sig.
Log Rank (Mantel-Cox)	,067	1	,796

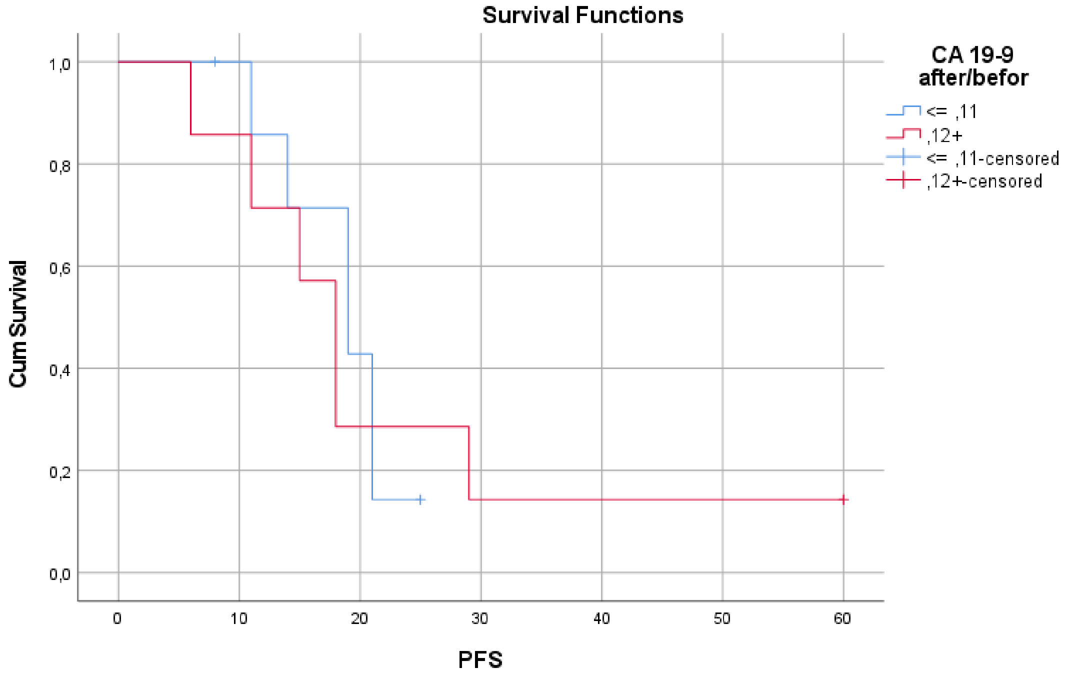

Supplement: Supplementary file 1 [file cancers-16-02234-s001.zip › Table S2. Statistics.pdf]
